# *In Silico* Structural Insights and Potential Inhibitor Identification Based on the Benzothiazole Core for Targeting *Leishmania major* Pteridine Reductase 1

**DOI:** 10.1021/acsomega.4c06146

**Published:** 2024-12-20

**Authors:** Jéssika de O. Viana, Karen C. Weber, Luiz E. G. da Cruz, Rhayane de O. Santos, Gerd B. Rocha, Alessandro K. Jordão, Euzébio G. Barbosa

**Affiliations:** † Department of Chemistry, Federal University of Paraíba, João Pessoa 58051-900, Brazil; ‡ Department of Pharmacy, Federal University of Rio Grande do Norte, General Cordeiro de Farias Street, CEP, 59012-570 Natal, RN, Brazil

## Abstract

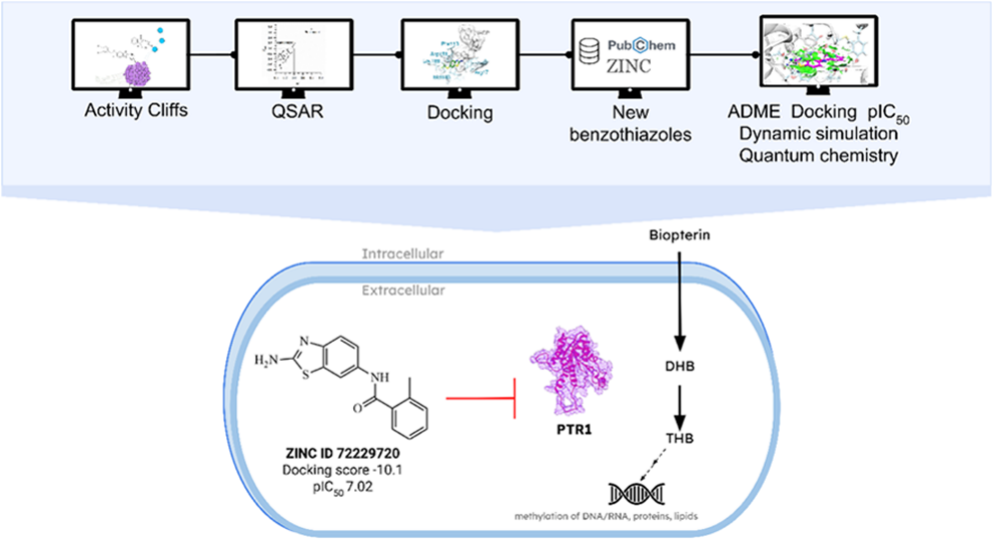

Leishmaniasis is reported as the second most common protozoonosis, with the highest prevalence and mortality rate. Among the Leishmania drug targets, Pteridine Reductase 1 of *Leishmania major* (*Lm*PTR1) proved to be promising because Leishmania is auxotrophic for folates. Thus, this study employed a combination of ligand- and structure-based approaches to screen new benzothiazole compounds as *Lm*PTR1 inhibitor candidates. Initially, a highly predictive quantitative structure–activity relationship (QSAR) model was able to identify the relevant hybrid descriptors, with an accuracy of over 93%. Insights into the mechanism of action indicated Phe113, His241, Leu188, Met183, and Leu226 as key residues. New commercially available compounds were screened using QSAR, docking, and pharmacokinetic properties as filters. Molecular dynamics, non-covalent interactions analysis, and quantum chemical calculation of binding enthalpy demonstrated that the lead compound (ZINC 72229720) forms a stable complex with *Lm*PTR1, indicating it as a new promising *Lm*PTR1 inhibitor.

## Introduction

Amidst all of the neglected tropical diseases, leishmaniasis stands out in particular. It is an infectious parasitic disease caused by intracellular protozoa of the *Trypanosomatidae* family, genus *Leishmania* spp.^1^ Moreover, it is one of the most common neglected diseases, and the need for research on new effective drugs to treat the manifestations of leishmaniasis is even greater.^2,3^

The existing first-line pharmacological treatments for leishmaniasis include pentavalent antimonials (meglumine antimoniate and sodium stibogluconate) and second-line treatments (amphotericin B, miltefosine, and paromomycin).^4,5^ However, current therapies have serious adverse effects such as abdominal pain, liver changes, cardiac symptoms, and toxicity, all of which are factors for treatment discontinuation and for the emergence of strain resistance.^2^

Designing new chemical agents with leishmania activity to address toxicity and resistance problems is pivotal to diminish patient suffering.^6^ To reduce the time and cost in medicinal chemistry, *in silico* tools are widely used for drug design.^7,8^ These strategies include computational studies based on ligand or receptor structures. In ligand-based drug design, structurally similar compounds that exhibit differences in activity can be evaluated using activity cliffs,^9,10^ identifying through them which small chemical modifications led to variations in the activity. In addition to this, other ligand-based techniques can be applied, involving the structural and physicochemical information on tested compounds that are used to train predictive QSAR or pharmacophore models, generating predictive models and highlighting the most important physicochemical characteristics for the targeted activity.^11^

On the other side, the receptor-based approaches include processes where information about the target and the ligand assists in interpreting the complex and also in searching for mechanisms of action. This method often involves several key techniques, such as comparative modeling, molecular docking, and molecular dynamics. The comparative modeling technique is used to predict the three-dimensional structure of a receptor when its exact structure is unknown, by using known structures of similar proteins as templates.^12^ Docking is a computational technique that simulates the interaction between small molecules and the receptor’s binding site to predict how well a compound might bind to the receptor.^8,13^ Molecular dynamics simulations provide a detailed view of the receptor’s behavior over time, offering insights into the flexibility and conformational changes of the receptor–ligand complex. The quantum chemical calculation of Δ*H*_binding_ is often a better approach to refine the docking results with a good correlation to experimental data.^14^ Together, these techniques enable a more precise design of compounds with improved efficacy and reduced off-target effects.^8,13^

**Figure 1:**
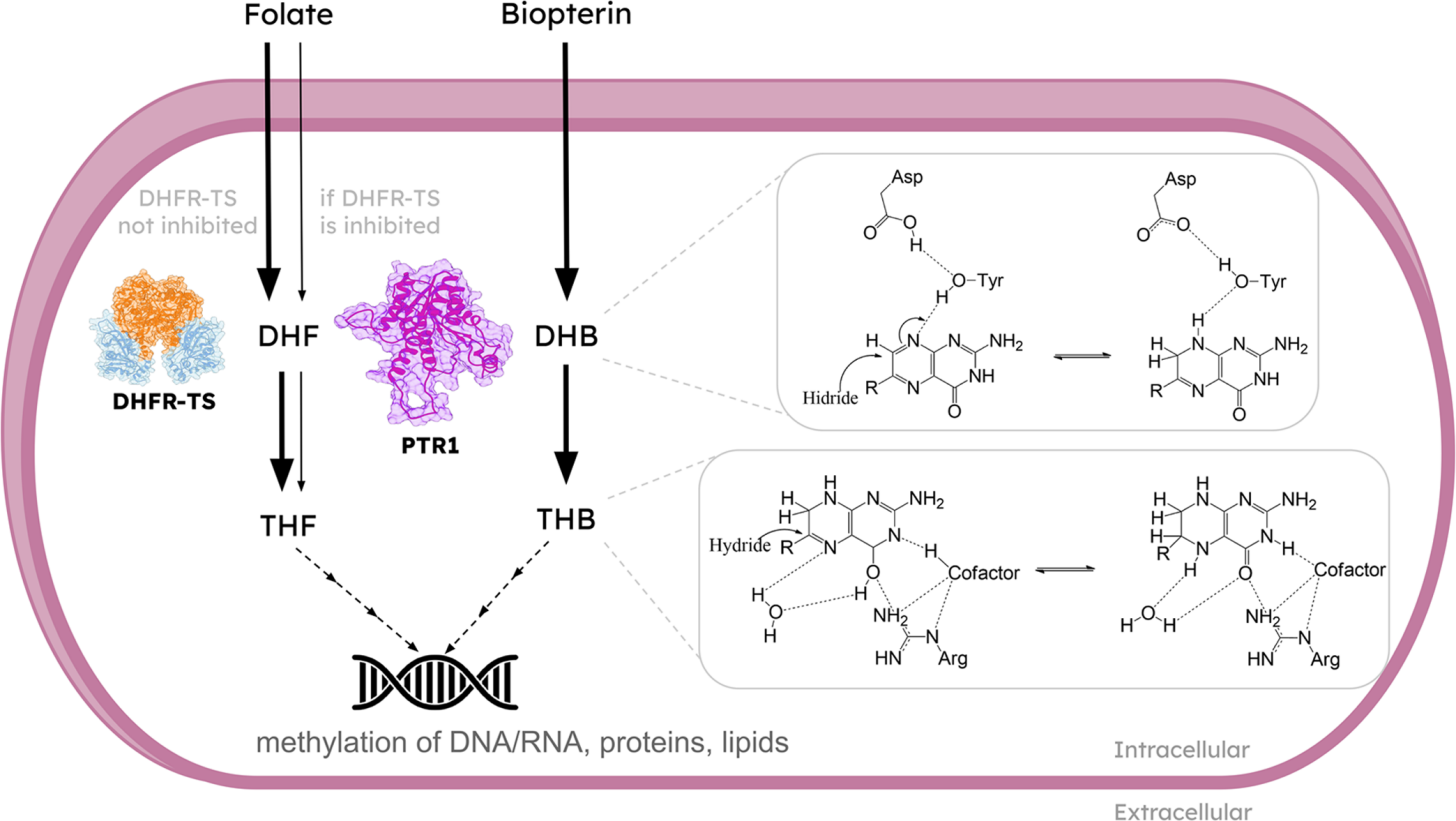
Metabolic pathway of folates and biopterin in *Leishmania* spp. The folate pathway primarily involves the DHFR-TS enzyme for the formation of dihydrofolate (DHF) and then tetrahydrofolate (THF). If the DHFR enzyme is inhibited, PTR1 provides sufficient folate to ensure the parasite’s survival. The biopterin pathway involves two steps where biopterin is first converted to dihydrobiopterin (DHB) and then DHB to tetrahydrobiopterin (THB). This reaction occurs through the interaction with Asp and Arg residues.

Studies have shown that with dihydrofolate reductase-thymidylate synthase (DHFR-TS) inhibition, pteridine reductase 1 (PTR1, EC 1.5.1.33) provides enough folate to guarantee the parasite survival.^15^ PTR1 is a member of the SDR (short-chain dehydrogenase/reductase) family and has proven to be essential in trypanosomatids by performing the reduction of folates and biopterins through a catalytic mechanism involving two distinct steps.^16^ After absorption, pterins such as biopterin and folate undergo two successive reductions to produce the tetrahydroactive species (Figure 1). In this process, two enzymes carry out these reactions in trypanosomatids: DHFR-TS is the primary enzyme responsible for reducing folate and 7,8-dihydrofolate (DHF) to 5,6,7,8-tetrahydrofolate (THF). On the other hand, PTR1 performs reductions of both conjugated and unconjugated pterins, for example, converting biopterin to dihydrobiopterin (DHB) and subsequently to 5,6,7,8-tetrahydrobiopterin (THB) or folate to DHF and THF when DHFR-TS is inhibited.^17^

It was observed that the PTR1 knockout gene proved to be lethal to the parasite probably because of the reduction in pterin levels produced only by PTR1. Pterin is essential for parasite growth and metacyclogenesis.^15^ Hence, PTR1 can be targeted to develop useful leishmanicidal drugs.^18^

New PTR1 inhibitors have been reported in the literature. For example, the study conducted by Istanbullu et al.^19^ evaluated a series of novel thiazolopyrimidine compounds with high antileishmanial activity against promastigotes of the species *Leishmania tropica* and *Leishmania infantum*. The core scaffold was created using different cyclic or acyclic tertiary amine groups at the 7-position of thiazolopyrimidine, which exhibited inhibitory activity against the enzyme *Lm*PTR1. However, the compounds demonstrated cytotoxic activity at the concentrations studied.

To shed light on this matter, a series of 69 benzothiazoles with *Lm*PTR1 inhibition were determined *in vitro* and published in the literature.^20,21^ These compounds have low or no cytotoxic activity, which helps in the optimization of new binders based on the structure of these derivatives. To identify the structural features of the existing inhibitors that could aid in the design of new inhibitors, the compounds were examined. The reported biological data were obtained for *Leishmania major*, which causes cutaneous leishmaniasis.

Activity cliffs and structurally similar clusters were explored here. Active inhibitors were selected to generate predictive QSAR models and docking studies. The structure–activity relationship and docking interaction features served as the basis for the design of new ligands against *Lm*PTR1, that were evaluated for the best absorption–distribution–metabolism–excretion (ADME) properties, prediction of IC_50_, molecular docking, molecular dynamics, and quantum chemical calculations.

## Results and Discussion

### Similarity Analysis and Activity Cliffs

**Figure 2:**
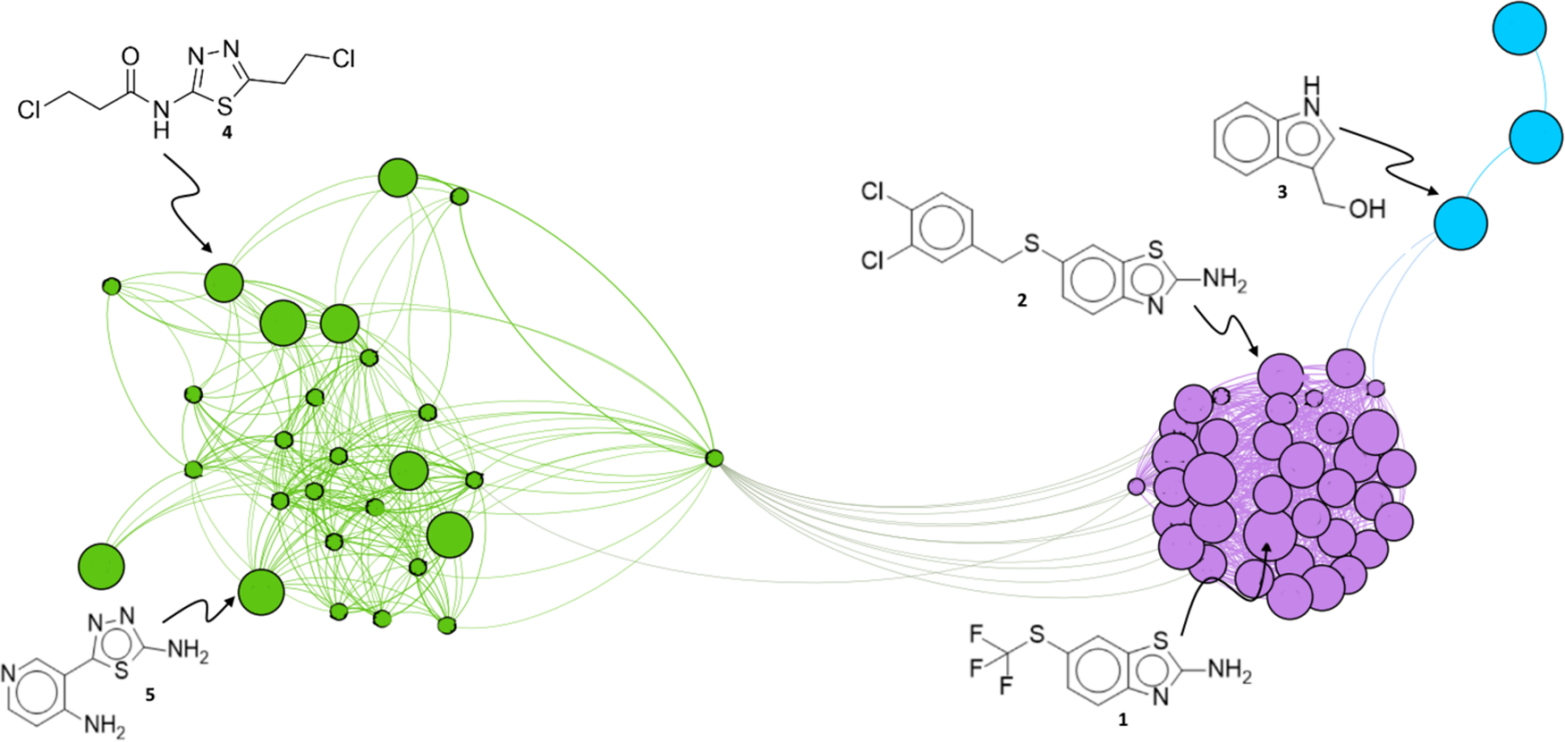
Clusters of structurally similar compounds plotted by Gephi software. The size of the nodes varies according to the inhibitory potency, and edges are weighted by TS. The three clusters obtained can be visualized by colors according to modularity statistics. The cluster in green is populated by compounds with the 1,3,4-thiadiazole (**4**, **5**) core and the purple cluster with the aminobenzothiazole core **(1, 2)**. In blue are some other less common cores **(3)**.

By analyzing the graph shown in Figure 2, it is possible to verify the formation of two main clusters that are differentiated by the compounds’ core. All inactive compounds do not contain the benzothiazole core in their structure, and when it is present, they have a methoxy group directly attached to the benzothiazole core. Figure 2 shows that the 1,3,4-thiadiazole core (green) produces analogues with lower efficacy, in contrast to the aminobenzothiazole core (purple). There are structures that are quite different from the others, which formed a small group (blue). This smaller cluster is shown to be populated by small potent compounds showing that the molecular volume does not necessarily reflect higher biological activity. Thus, it is necessary to optimize the interaction with regions other than those where the ligand performs π–π stacking with the NAD.

**Figure 3:**
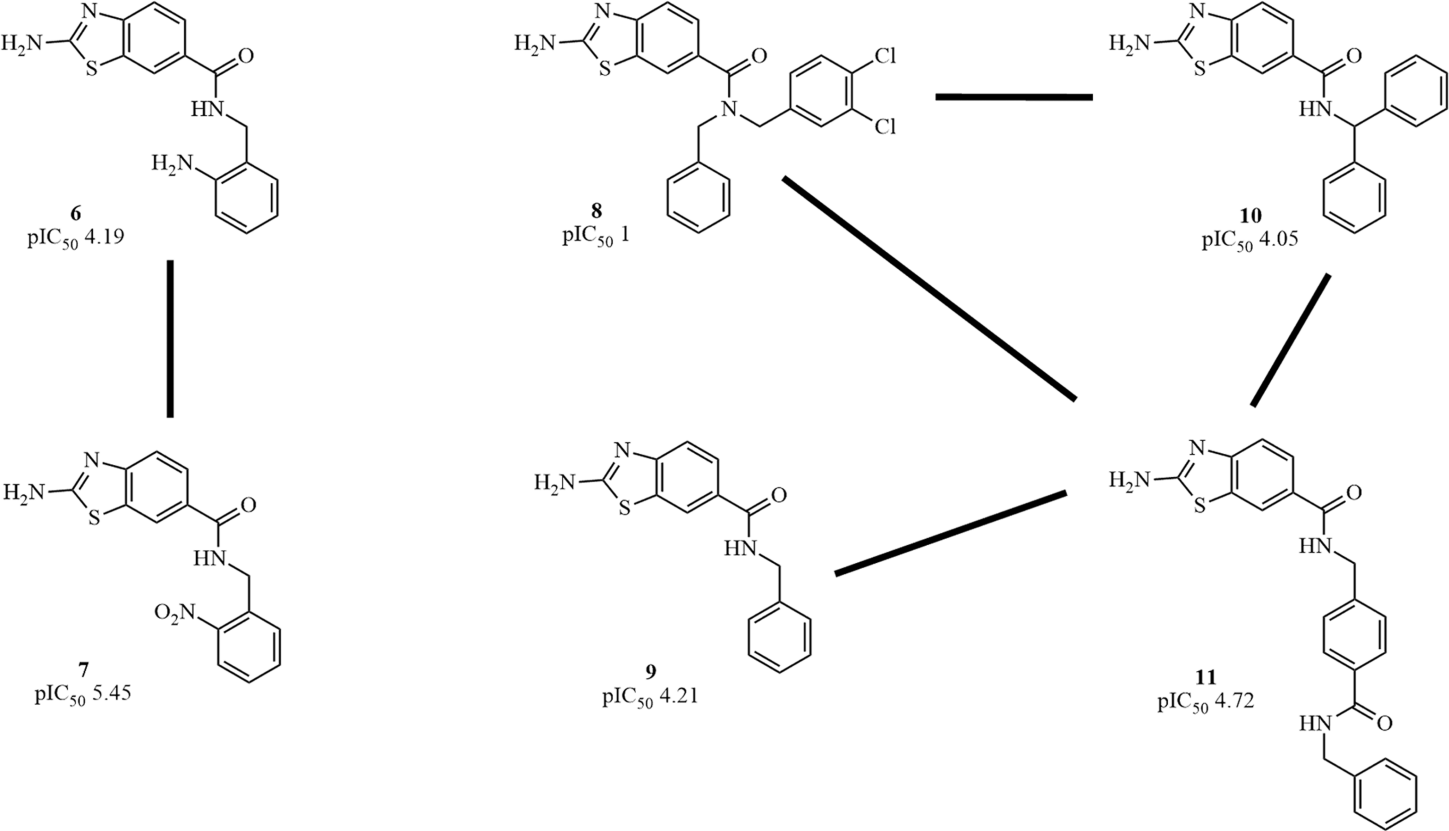
Activity cliff of compounds obtained by filtering the SALI. The IC_50_ value, reported in previously published works,^20,21^ was used to calculate the pIC_50_.

Activity cliff analysis helps to define moieties that are responsible for large differences in biological activity. In Figure 3, it is possible to verify that compounds **6** and **7** showed that the nitro group has a better profile than the amine portion. It can also be noted that the introduction of fragments to the amide portion can increase or decrease the affinity with *Lm*PTR1. Compound **8** (inactive) presents two aromatic ring systems that are more widely spread compared with compound **10** (active). This property may arise from a hindered inclusion in the *Lm*PTR1 site.^21^ Smaller and straighter moieties are better suited to interact with *Lm*PTR1, as demonstrated by compounds **9**, **10**, and specially **11** (Figure 3).

### *Lm*PTR1 Present a Similar Active Site to *Tb*PTR1

In order to determine the position of compound 1, the most active compound of the series, in the active site of *Lm*PTR1, we searched the Protein Data Bank (RCSB PDB) for resolved three-dimensional structures. We observed that there is no resolved three-dimensional structure of the *Lm*PTR1 complex with compound 1. However, there is crystallographic data for the enzyme *Tb*PTR1 with compound 1 (PDB ID 6GCQ).^21^ Due to the lack of crystallographic data for the benzothiazole core in the *Lm*PTR1 enzyme, a superposition of the *Lm*PTR1 enzyme (PDB ID 5L4N) with *Tb*PTR1 (PDB ID 6GCQ) was performed to aid in the investigation of interactions and the position of benzothiazole derivatives in the catalytic site.

The superposition of *Lm*PTR1 with *Tb*PTR1 showed an RMSD of 0.539 Å. It was identified that the active sites of the enzymes *Lm*PTR1 and *Tb*PTR1 are similar, with differences only in 4 amino acid residues: Ser112Ala, Leu118Cys, Leu226Val, and Val228Leu (Figure S1). The exchange of these amino acid residues suggests a change in hydrophobicity in the structure of the active site, which can indicate the difference in the activity of the compounds *in vitro* on the enzymes *Lm*PTR1 and *Tb*PTR1, as demonstrated by Linciano and co-workers.^21^ In another work, it is possible to note that *Tb*PTR1 inhibitors interact similarly with those of *Lm*PTR1, where the residues are conserved between the species, especially Arg14, Ser95, Phe97, Asp161, and Tyr174 were conserved among all of the trypanosomatids.^22^

### QSAR Models Present High Predictive Power

**Figure 4:**
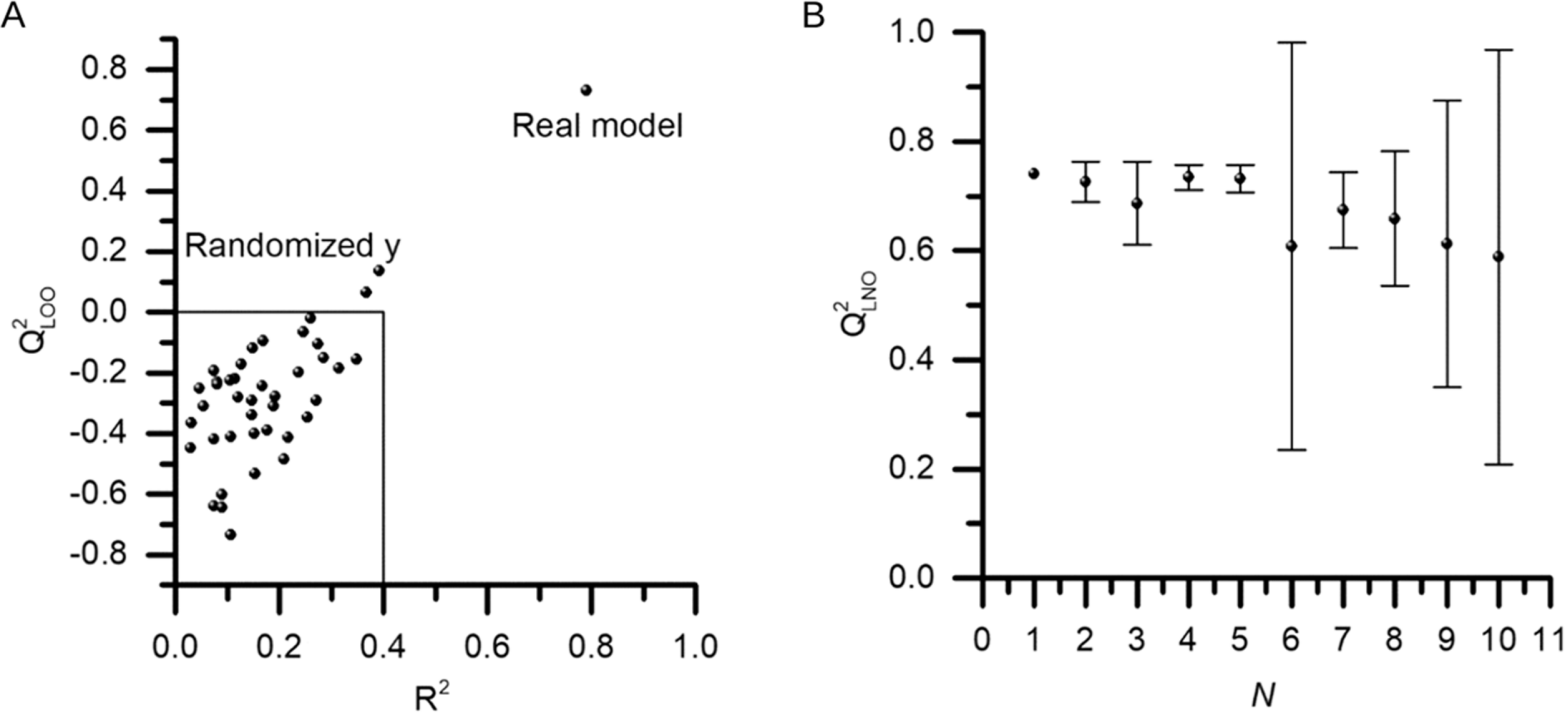
QSAR model results. (A) *y*-randomization validation, where models built with randomized *y* proved to be very poor compared to real model figures of the real model; (B) LNO validation applied to models with the removal of up to 10 samples. *Q*_LNO_^2^ produced models comparable to the *Q*_LOO_^2^ value. The LNO validation was performed 10 times for each *N*, and *Q*_LNO_^2^ deviations are shown around the mean value.

QSAR models were built using 34 compounds in the training set and 11 compounds in the test set. The best results obtained using the two-dimensional (2D) descriptors presented *R*^2^ = 0.79, *Q*_LOO_^2^ = 0.73, and *Q*_ext_^2^ = 0.68. Similar results were observed for the three-dimensional (3D) model (*R*^2^ = 0.79, *Q*_LOO_^2^ = 0.61, and *Q*_ext_^2^ = 0.61). The best QSAR model performance was obtained using a combination of 2D and 3D descriptors (*R*^2^ = 0.93, *Q*_LOO_^2^ = 0.95, *Q*_ext_^2^ = 0.89, Table S1 and Figure S2a,b). This hybrid model had a better performance for the training set and test set when compared to isolated models in 2D or 3D descriptors (Table 1). Also, the hybrid model used a total of four 2D descriptors and three 3D descriptors with 11 latent variables (Figure 4).

**Table 1:** Determination Coefficient (*R*^2^) and Predictive Power (*Q*_ext_^2^) for All of the Models Using 2D and 3D Descriptors, and a Hybrid Approach

models	*R*^2^	*Q*_LOO_^2^	*Q*_ext_^2^
2D	0.79	0.74	0.69
3D	0.79	0.61	0.62
hybrid (2D + 3D)	0.93	0.95	0.89

All models were validated through an LNO test to prove their robustness (Figure 4A). For this analysis, a total of 10 samples were removed, which proves that robust models were obtained with no major reductions in the *Q*_LNO_^2^ values (*N* = 5, Figure 4B). Additionally, the y-randomization tests showed that the models were not obtained by chance, as the real models have far better figures of merit compared to models generated with randomized pIC_50_ values (Figure 4A). Also, the correlation matrix of the molecular descriptors did not find highly correlated descriptors in the study (Figure S2).

The 2D part of the model ATSC7s represents the contribution of the charge of the electronegative atom in the fragment coupled to the 2-aminobenzothiazole moiety next to the His241 amino acid residue in the active site (Figure S3). These characteristics are present in the most active compounds. Likewise, the descriptor GATS 5p favors the contribution of the weighted by polarizability in chlorine charge in the fragment 2-aminobenzothiazole, also next to the adjacent His241.

The Burden-modified eigenvalue descriptor is related to the Burden matrix weighted by mass in the largest eigenvalue number 7 (SpMax7_Bhm), and it can be correlated to the molecular weight of the molecule. At this point, we note that the positive sign indicates that an increase in the overall molecular weight of the compound increases the pIC_50_ values of the compounds. Moreover, the descriptor ndssC is related to the number of atoms of the type dssC, where there is encoded information about the electrophilic characteristics of these compounds. These descriptors were distributed to the regions of Tyr283 and His241 residues, where electrophilic contact with these residues was present in more active compounds (Figure S3).

Regarding the 3D descriptors, it is observed that the HF descriptor detects the presence of hydrophobic moieties at a particular volume. The presence of the HF descriptor was observed in the 2-aminobenzothiazole core, during interaction with the Phe113 residue and NADP related to π–π interactions in this region (Figure S3). On the other hand, the LJ descriptor detects the presence of atoms in a particular space volume or refers to middle-range favorable interactions, such as a binding pocket. This descriptor demonstrates that compounds with fragments coupled to the 2-aminobenzothiazole core had better activity. The presence of these groups in the binding pocket can be related to better or worse interactions with His241 and Leu226.

### Molecular Docking of Benzothiazole Derivatives in *Lm*PTR1

The *Lm*PTR1 structure was superposed to the *Tb*PTR1 one and redocking of compound **1** in the active site of *Lm*PTR1 was performed. This analysis proved to be validated by comparison with compound **1** crystallized binding pose at *Tb*PTR1 (RMSD 0.55 nm, Figure S4). Additionally, the ROC analysis presented an AUC (area under curve) of 0.913, with a BEDROC of 1 (Figure S5), indicating a good prediction of the model.

**Figure 5:**
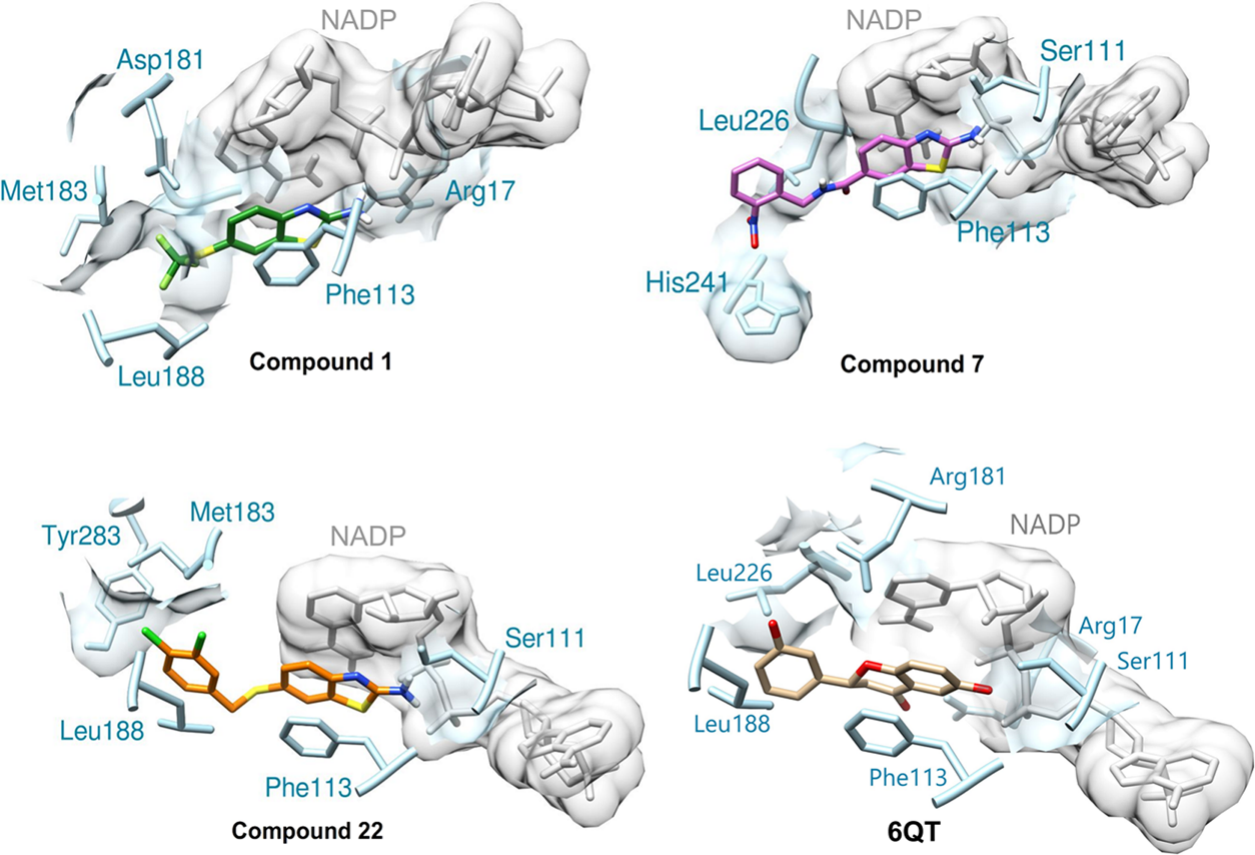
Representation of the molecular docking results in *Lm*PTR1 (PDB ID 5L4N, blue) and the NADP in gray color. The three best compounds (**1**, in green, **7** in pink, and **22** in orange) and the reference ligand (PDB ID 6QT) are presented in the active site of the enzyme, along with the amino acid residues that interact with each compound.

After validation, interactions of the benzothiazole derivatives in the *Lm*PTR1 active site were investigated. The compounds present the benzothiazole core superposed on the core of compound **1**. The most active compounds in the series (compounds **1**, **7**, and **22**), based on IC_50_ values, also exhibited the best score values (Table S2). The benzothiazole group forms hydrogen bonds with NADP and Ser111, while π-type interactions stand out with residues Phe113, His241, Leu188, Met183, and Leu226 (Figure 5). These residues are similar to those found for the reference inhibitor (6QT), as hydrogen bonds with Ser111 and Arg17 in the hydroxyl and ketone fragments and the aromatic groups enabled the formation of pi-stacked interactions with Phe113, Leu188, and Leu226 (Figure 5),^24^ indicating that the compounds exhibit a similar interaction mechanism with the protein. These observations were also found by Linciano et al.,^21^ where the benzothiazole core formed a sandwich between the cofactor and Phe113.

Structures similar to those of the benzothiazole group have been studied in the literature. For example, Mpamhanga et al.^23^ identified a series of aminobenzothiazole and aminobenzimidazole compounds as inhibitors of *Tb*PTR1. Compounds containing the aminobenzothiazole core exhibited apparent *K*_i_ (*K*_i_^app^) values of approximately 21 and 141 μM against *Tb*PTR1. In molecular docking studies, the ligands were found to be sandwiched between the aromatic moiety of Phe97 and Tyr174 (the same residues in *Lm*PTR1 are Phe113 and Tyr191) and the nicotinamide part of NADP^+^, as well as forming a hydrogen bond with Ser95 (the same residue in *Lm*PTR1 is Ser111) and two with the cofactor. These results were similar to what we found in our study for *Lm*PTR1, indicating the similarity in the interaction that benzothiazole derivatives have in inhibiting the PTR1 enzyme of trypanosomatids.

Another example is the study by Istanbullu et al.,^19^ where compounds bearing chlorine or nitro substituents on the benzamide part of the thiazolopyrimidine-benzamide scaffold exhibited high antileishmanial inhibitory activity. The best compounds also showed high activity against *Lm*PTR1, with the top compound exhibiting an IC_50_ of 6.02 μg/mL as the most promising candidate.

### New Inhibitors and the Potential Activity in *Lm*PTR1

These analogues were searched based on the 2-aminobenzothiazole core, present in the most active compound in the series. After searching 1394 compounds, they underwent ADME properties calculation. Out of these, a total of 493 compounds were filtered based on high gastrointestinal absorption (GI), log *P*_o/w_, solubility using Ali class (soluble and moderately soluble), no BBB permeation, no P-gp substrate, and drug-likeness according to Lipinski, which establish standards to ensure good oral bioavailability. After the search, the filtered compounds were simulated in the active site of *Lm*PTR1.

**Figure 6:**
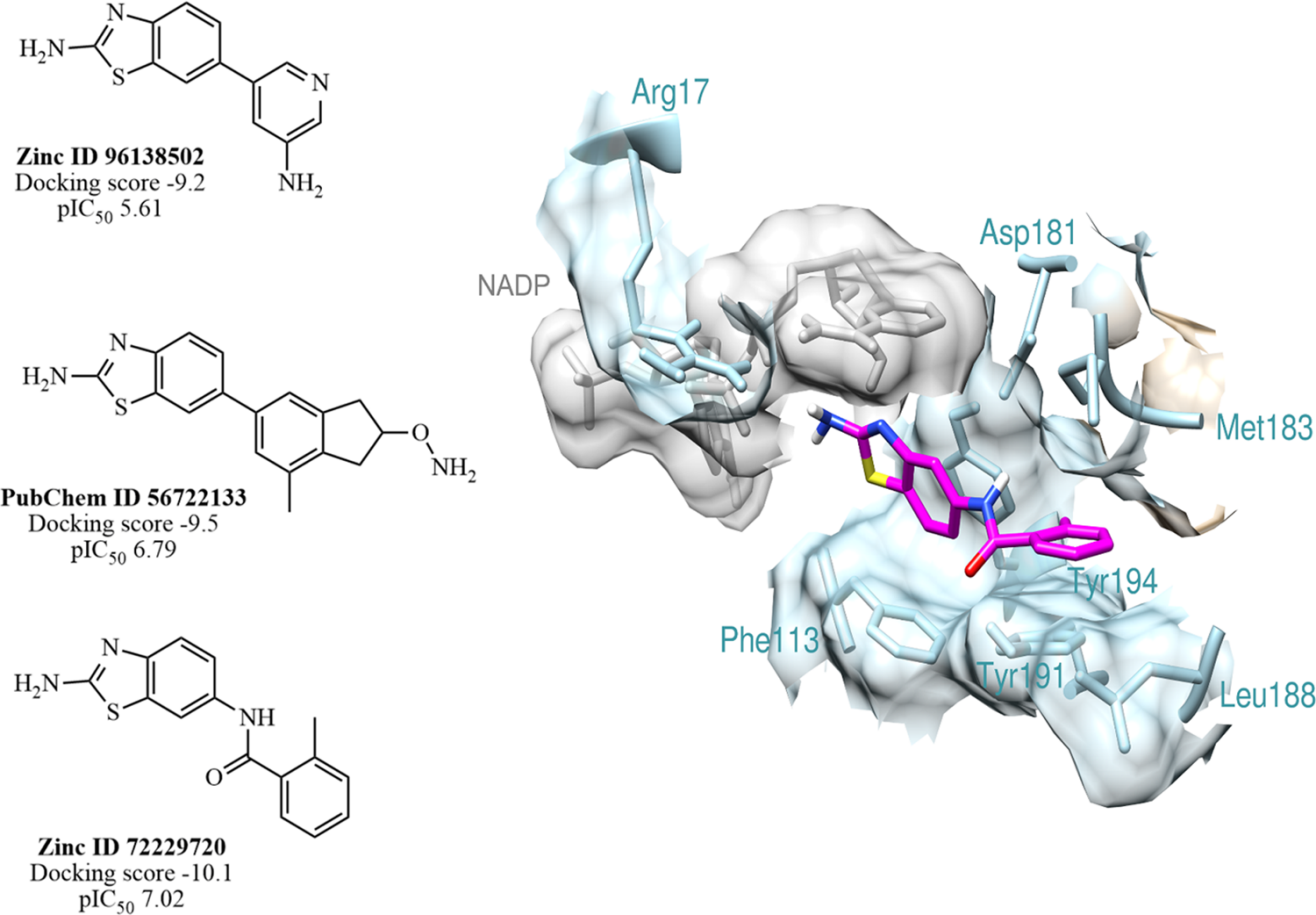
Three best analogues screened as *Lm*PTR1 (PDB ID 5L4N) inhibitors, with their docking score and QSAR estimating biological activities (pIC_50_) and docked conformation of ZINC 72229720 in the active site of *Lm*PTR1. The protein is highlighted in blue, the NADP in gray, and ZINC 72229720 in pink.

Among the evaluated compounds, we performed structural and electronic comparisons with the entire ZINC and PubChem databases, leading to the identification of three commercially valuable compounds: PubChem 56722133, ZINC 72229720, and ZINC 96138502. The consensus analysis of docking scores, pIC_50_, and ADME features for these compounds indicate that the potency against *Lm*PTR1 may be higher than that of compound **1**, mainly for the ZINC 72229720 with a pIC_50_ value of 7.02 (Figure 6). The Tanimoto coefficients of the best compound, ZINC 72229720, were compared with the benzothiazole derivatives from this study (Table S3), and it was observed that the highest similarity was found with compound 2 from the series (0.83).

When we analyze the docking poses of ZINC 72229720 in the active site, we found the interaction of the Phe113 amino acid residue within the aromatic ring of the analogues, supported by an additional π–π interaction (Figure 6). In addition, the presence of heterocyclic groups coupled to the 2-aminobenzothiazole core can positively interact with the Leu226 amino acid residue in the active site of *Lm*PTR1, where the analogue conformation to the active site resembles the conformation of compound **1**. A positive hydrophobic characteristic is observed in the interaction of the aliphatic portion with Met183, Leu188, and Tyr194 residues, which favors the interaction in the active site.

Similar interactions were observed by Di Pisa et al.^24^ using a series of chroman-4-one derivatives as inhibitors of *Lm*PTR1, with the best inhibitor presenting an IC_50_ value of 57 μM (PDB ID 5L4N). In this assay, the molecular docking and crystallography studies identify similar conformations of chromenone and confirm Leu188 and Leu226 as critical interacting residues in the active site. Additionally, Shtaiwi et al.^25^ also found that Phe113, Leu188, and His241 make π interactions with aromatic groups using a series of thiadiazine-thiones derivatives. These studies indicated that the benzothiazole core presents similar positions compared with chromenone and thiadiazine-thiones cores.

### Stability of Complex *Lm*PTR1 and ZINC 72229720

To thoroughly evaluate the behavior of the *Lm*PTR1-ZINC 72229720 complex, a molecular dynamics study was conducted. For comparison, the chroman-4-one inhibitor (PDB ID 5L4N, ligand ID 6QT(24)) was used. In molecular dynamics, we observed the stability of the ligands and the protein at the catalytic site along the trajectory (Figure S6a,c). The stability of the *Lm*PTR1-6QT complex is achieved through π interactions between the Phe113 and Leu188 residues with the chroman-4-one moiety, in addition to hydrogen bonds between this group and the Ser111 and Arg17 residues (Figure S6e). Similarly, the complex with *Lm*PTR1-ZINC 72229720 indicated the presence of one stable conformation in the complex (Figure S6b,f), where the benzothiazole core presents interactions with amino acid residues Phe113 and NADP, through π interactions, and hydrogen bonds between Ser111 and NADP (Figure S6d). Thus, the methylbenzene group preserved the π interactions with Met183 and Leu188, indicating the importance of this group for complex formation.

Comparing the ADME properties, ZINC 72229720 presents a better lipophilicity (log *P*_o/w_) than compound **1** and better pharmacokinetics (no P-gp substrate) than the 6QT inhibitor, which is pointed as a P-gp substrate (Table S4). In this way, the benzothiazole group presented itself as a structurally viable core for interaction, proving to be a good prototype for the design of new *Lm*PTR1 inhibitors.

### Non-Covalent Interactions (NCI) Results

**Figure 7:**
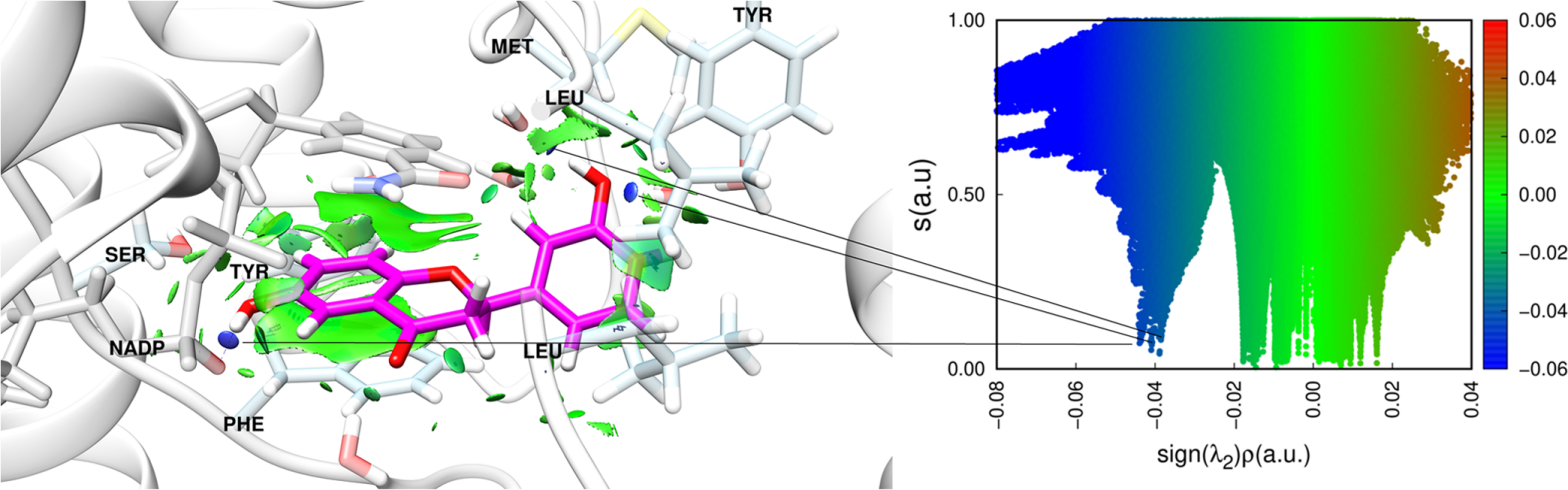
3D representation of non-covalent interactions from promolecular density. Right panel shows the graph of density gradient and the product of lambda and density for PBD 6QT, a ligand of PDB ID 5L4N.

According to the NCI calculations, as shown in Figure 7, three hydrogen bonds are crucial for maintaining the ligand in the active site. Among these bonds, two are formed with water molecules and one is formed with the NADP cofactor. The interaction with NADP is the most attractive and therefore the most important for keeping the inhibitor on the active site, as evidenced by a peak located further to the left of the graph.

Another significant interaction occurs between the plane of the inhibitor’s aromatic ring in the PDB and the Phe113 residue, as well as the aromatic ring present in NADP. This result aligns with findings in the literature,^25^ which highlight the importance of these interactions, suggesting that other *Lm*PTR1 inhibitor candidates should exhibit similar interactions.

**Figure 8:**
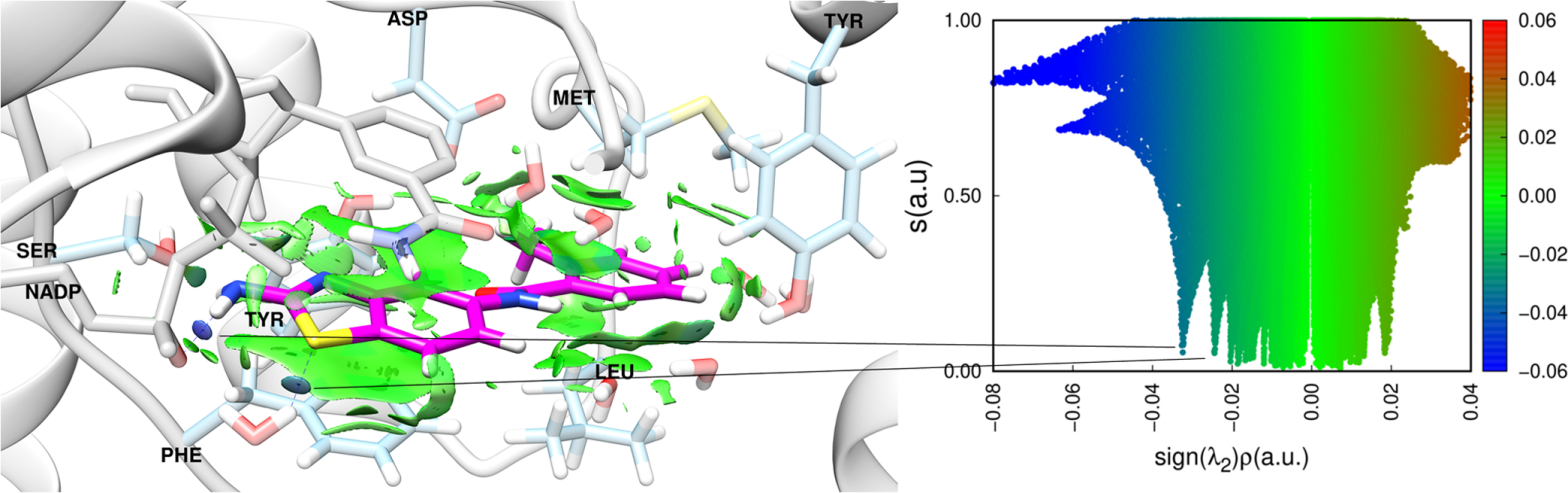
3D representation of non-covalent interactions from promolecular density. Right panel shows the graph of density gradient and the product of lambda and density for ZINC 72229720.

As proposed in Figure 7, the results in Figure 8 confirmed the previously raised hypothesis. The most important attractive interaction observed with NADP was repeated, once again appearing as the blue peak furthest to the left of the graph. However, only one bond was formed between a water molecule and the sulfur atom in the ZINC 72229720 ligand. This shows that, for this ligand, direct interactions with residues in the active site may be more important for maintaining the ligand stability in the protein. This also indicates that the PDB 6QT ligand is more susceptible to solvation effects than the ZINC 72229720 ligand.

Furthermore, the results suggest the existence of two regions capable of interacting with the rings present in the ligand. One of these interactions occurs in the ligand between the NADP ring and the Phe113 residue, while the other occurs between the residues of Leu188. These findings indicate that the ligand has new types of interactions with the protein residues, yet its stability is maintained.

**Figure 9:**
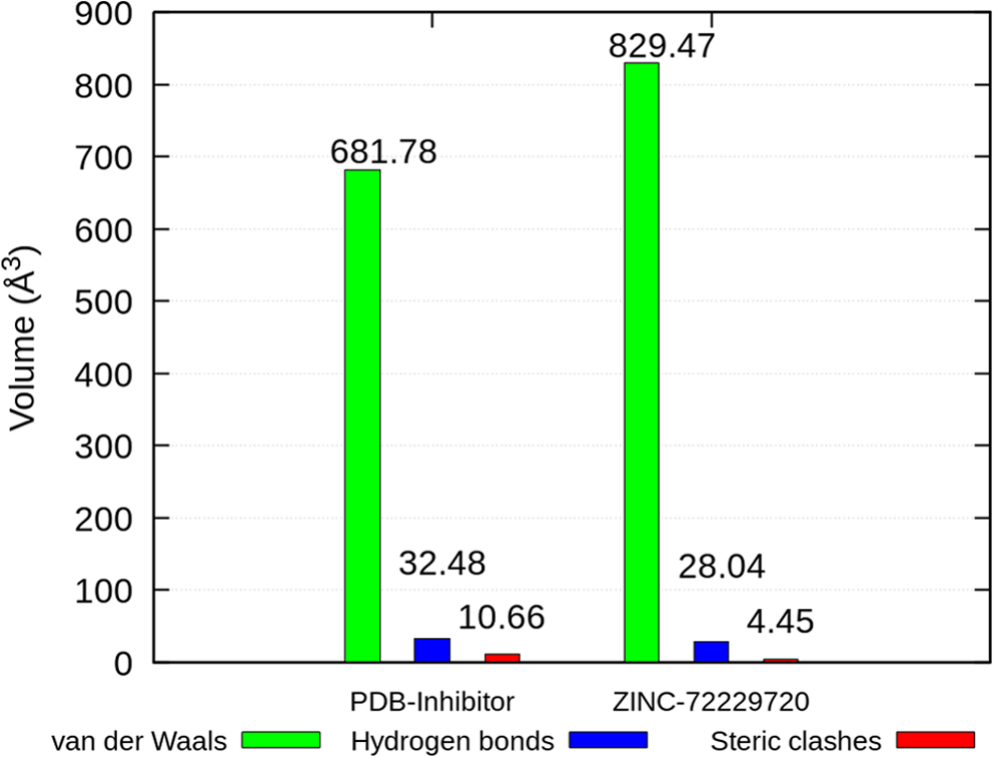
Integral of the volumes of the interaction surfaces resulting from the NCI calculations.

Overall, it is evident that there is a significant difference in the occurrence of each type of interaction for each ligand. Although the volumes of the isosurfaces for hydrogen bonds are small, they represent the most attractive interactions. In this regard, Figure 9 shows that the PDB 6QT ligand exhibited a higher value (32.48 Å^3^) compared to the ZINC 72229720 ligand (28.04 Å^3^), as previously shown in the emergence of hydrogen bonds.

In terms of van der Waals interactions, a notable discrepancy between the two values was observed (Figure 9). The ZINC 72229720 ligand showed the highest value (829.47 Å^3^), suggesting that the sum of these weaker interactions may compensate for the lower value of stronger interactions relative to the PDB entry 6QT ligand. Furthermore, the ZINC 72229720 ligand presented a lower value of repulsive interactions. These results strongly indicate that the ZINC 72229720 ligand could be a notable inhibitor of the *Lm*PTR1 enzyme.

### Quantum Chemical Calculation of Δ*H*_binding_

Through the calculation of binding enthalpy using the PM7 method, the following values were obtained: for ligand PDB 6QT, Δ*H*_binding_ = −39.28 kcal mol^–1^ and for ligand ZINC 72229720, Δ*H*_binding_ = −36.14 kcal mol^–1^. The difference between these two values is small, suggesting that both have significant potential for enzyme inhibition. Therefore, ligand ZINC 72229720 is indeed a promising candidate to replace ligand PDB entry 6QT, provided it offers additional advantages such as fewer side effects, lower production costs, and improved pharmacokinetic properties.

## Conclusions

In this study, we found that the results presented in the activity Cliff, QSAR, and docking analyses represent similar interpretations that help to better understand the effects of biological activity. Hybrid descriptors showed better predictive values (93%) compared to those of 2D or 3D unit descriptors. These results helped in the interpretation and identification of crucial features to distinguish the more active and less active compounds, such as the topological charge, Barysz matrix between heteroatoms, Lennard-Jones potential, and hydrophobic and hydrogen bonding, which are essential for the activity of benzothiazoles in *Lm*PTR1. Furthermore, molecular docking presents a validated protocol with an AUC of 91% prediction in the correct score of active compounds versus inactive compounds. Still, docking indicates key residues included in the benzothiazole core. Based on these models, the virtual screening for new benzothiazole derivatives indicated that the compound ZINC 72229720 showed excellent biological prediction, docking score, and ADME properties, better than the PDB inhibitor. Furthermore, molecular dynamics simulations showed stability in the formation of complexes with the *Lm*PTR1 enzyme. Finally, NCI calculations revealed the emergence of new interactions between the ZINC 72229720 ligand and the enzyme, resulting in a better balance between the forces that stabilize the ligand in the active site. Binding enthalpy calculations showed that the ligand is a promising inhibitor for *Lm*PTR1.

## Methods

### Data Set Selection

The *Lm*PTR1 inhibitors structural data set was built from literature data (Table S5).^20,21^ All compounds exhibited a defined stereochemistry. Among 69 compounds, 24 were reported inactive and 45 have defined biological activity ranging from 1.9 to 1800 μM. To build the QSAR models, the pIC_50_ values, [−log(IC_50_)], were used to calibrate the vector of biological activity (*y*). Molecular models of all compounds were created using MarvinSketch 19.21 (ChemAxon), considering a pH of 7.40, to estimate the best microspecies to simulate physiological conditions. All models were converted to 3D coordinates and carefully curated by visual inspection using Avogadro software.^26^ Molecular geometries were optimized with the PM7 semiempirical method in MOPAC^27^ using the implicit COSMO solvation model.^28^

### Structural Comparison of *Lm*PTR1 and *Tb*PTR1

Due to the lack of *Lm*PTR1 crystallographic data with the benzothiazole core, a superposition of *Lm*PTR1 (PDB ID 5L4N(24)) with *Tb*PTR1 (PDB ID 6GCQ(21)) was done to investigate the inhibitor’s interactions and to aid molecular alignment (Figure S7). The structure of *Tb*PTR1 was chosen because one of the benzothiazole derivatives (compound 1) is present in the active site. The 3D structure of this target was aligned using UCSF Chimera,^29^ containing inhibitor **1** (compound **1**) in the catalytic site. The superposed structure of *Lm*PTR1 was saved in the PDB format.

### Molecular Alignment of Benzothiazole Derivatives

To calculate the 3D-QSAR descriptors, it is imperative to align all molecular models to their biologically active conformations. Inhibitor **1** (compound **1**), the most potent compound of the benzothiazoles series, was used as a template to align all remaining 44 active compounds from the series.

The molecular alignments were performed automatically using *gmx confrms*, the module embedded in the simulation package GROMACS.^30^ The most appropriate sequence of atoms was selected by visual inspection using UCSF Chimera software.^29^ The 2-aminobenzothiazole moiety was used as the standard for the alignment (Figure S8). Compound **1** served as a reference for the alignment of the other compounds. Once all molecules were aligned, they were visually inspected and sometimes slightly adjusted using UCSF Chimera^29^ to ensure that the supposed bioactive conformations did not collide with the adjacent side chains of the *Lm*PTR1 binding site.

### Activity Cliffs

Activity cliff analysis^31^ was performed to identify significant differences in biological activity caused by small changes between similar structures. For this analysis, we used the complete set of 69 compounds. OpenBabel^32^ was used to calculate the Tanimoto Similarities (TS)^33^ between all compounds. Based on the TS, the Structure Landscape Activity Index (SALI) was calculated,^34^ as shown in eq 1
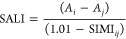
1where *A_i_* and *A_j_* represent the pIC_50_ of two compounds, and SIMI*_ij_* is TS between compounds *i* and *j*.

To interpret the data, two types of graphs were created using the Gephi software.^35^ The first graph connects compounds according to their respective TS, where the edge weights are similar and the nodes are scaled by one compound potency. Edges with similarity values lower than 0.6 were removed to allow for the formation of clusters. The second graph was created by employing high SALI values as edge weights.

### QSAR Descriptors Calculation

To calculate the necessary molecular descriptors, only the compounds showing inhibitory activity for the PTR1 enzyme, 45 compounds in total, were considered. Two programs were used to calculate the 2D and 3D descriptors. The software PaDEL^36^ was used to generate the 2D descriptors with the same aligned compounds. The 3D descriptors were computed using our *ad hoc* software written in Python: 3D-QSARpy.^37^

The 2D descriptors were filtered prior to variable selection. Columns with missing values, invariant vectors, and those with high intercorrelation (above 0.98) were removed according to the protocols described in the literature.^38^ The described filtering process was performed using R (version >3)^39^ and the Caret package.^40^

3D-QSARpy computes four types of descriptors using a sp^3^ + 1 charged carbon atom going through a lattice with 1 Å spacing adjusted around the aligned structures. The descriptors are (i) van der Waals descriptors computed by the Lennard-Jones equation (LJ), (ii) Coulomb descriptors (QQ), (iii) descriptors that detect the presence of groups that can form hydrogen bonds (HB), and (iv) descriptors that detect the presence of hydrophobic groups (HF). The parameters used to calculate the Lennard-Jones descriptors were derived from the MMFF94 force field. Atomic charges were calculated by using the Gasteiger method. The detection of the presence of groups was performed using Gaussian curves centered on the nuclei of the O and N atoms for (HB) and on the non-O and non-N atoms for (HF) (Table 2).

**Table 2:** 3D Descriptors Were Calculated for the Construction of Models^a^

descriptor	formula
LJ (Lennard-Jones)	
QQ (Coulomb electrostatic interactions)	
HB (hydrogen bond forming atoms)	*A* e^–10(*r*–1)^2^^
HF (hydrophobic atoms)	*B* e^–10(*r*–1)^2^^

a The parameters ε and σ are the types of combined atoms derived from the MMFF94 force field, ρ is the middle dielectric constant (ρ = 1), and *r* is the distance between the interacting atoms. *A* = 1 for interactions with O and N. *B* = 0 for atoms of O and N.

The 3D descriptor matrix was filtered by removing descriptors with a variance less than 0.01 and intercorrelation greater than 0.95. The filtered descriptor matrix was subjected to variable selection methods described as follows.

### QSAR Models

Various QSAR models were created by employing only 2D descriptors, 3D descriptors, and a combination of both types of descriptors to predict *Lm*PTR1 activity. First, we used the ordered predictors selection approach (OPS)^41^ to reduce the dimensionality of the data. Then, the OPS output file was used to build the linear regression model of the independent variables using the stepwise multiple linear regression (S-MLR) method implemented on NanoBRIDGES software.^42^

Subsets of compounds were selected to assemble a training set and a test set to evaluate the predictive power of each QSAR model (70 and 30% of the total 45 structures, respectively). The sets were selected based on molecular diversity and activity range (Table S5). The models were considered robust for prediction only when *R*^2^ > *Q*_LOO_^2^, *Q*_ext_^2^ was higher than 0.70.^43^

To evaluate whether the models were obtained by chance, the y-randomization^44^ test was performed. In this analysis, models with randomized biological activity are built and the *R*^2^ and *Q*_LOO_^2^ values must be much lower than the actual model figures of merit. To evaluate the models’ robustness, the Leave-N-Out (LNO) cross-validation test was used. Models were built by gradually removing *N* samples up to 50% of the total number, and the values of *Q*_LOO_^2^ were evaluated.^43^ The test results were evaluated comparing *Q*_LOO_^2^ and *Q*_LNO_^2^.^45^

A correlation matrix of the molecular descriptors was prepared using a python script, and highly correlated descriptors with a correlation value of 0.9 or above were removed from the study.

### Molecular Docking of Benzothiazole Series

Initially, redocking was performed on the *Lm*PTR1 protein (PDB ID 5L4N(24)). The crystallized inhibitor **1** was used as a reference and redocked into the active site by using a 20 Å grid box. The root mean square deviation (RMSD) value between the position of the crystallized inhibitor 1 and its redocked pose was calculated and considered only when below 2 Å. The protein structures underwent preparation steps, including solvent deletion, addition of hydrogens, charges, and replacement of incomplete side chains using the UCSF Chimera program.^29^ The grid box was marked with a 20 Å^3^ grid around the inhibitor PDB on the active site. Docking was performed using the Vina program, utilizing the virtual screening script, and the interactions were analyzed in UCSF Chimera.^29^

After redocking validation, molecular docking of all compounds was executed and a second validation was performed. Of the 69 compounds from the data set, 45 were classified as active and 24 as inactive compounds (Table S5). All of them were docked into the active site of the *Lm*PTR1 target (PDB ID 5L4N(24)) using the same redocking protocol. Docking was performed using the Vina program employing the virtual screening script, and the interactions were analyzed in UCSF Chimera.

Then, the docking scores were compared with the inhibition values of the compounds on the *Lm*PTR1 enzyme for a second validation.^46^ The docking score was used, and a second variable was created using a value of 1 for active compounds and a value of 0 for inactive compounds. The AUC of the ROC curve was calculated using the server https://stats.drugdesign.fr/,(47) estimating that the closer to 1, the better the prediction of the model.^48^

### Design of New Benzothiazole Inhibitors

**Figure 10:**
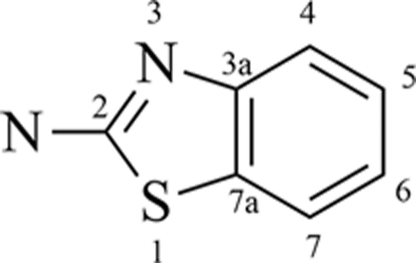
Chemical structure of the template core with the IUPAC number, indicating where new insertions produced the designed library.

The 2-aminobenzothiazole core (Figure 10) was compared with other commercially available compounds using the Swiss-similarity^49^ tool (ZINC Lead-like, scaffold) and by 3D structural comparisons with the Pubchem database.^50^ In Pubchem, a total of 992 similar compounds were found and 400 similar compounds using SwissSimilarity.

To analyze the *in silico* pharmacokinetic profile of the commercial analogues and compound **1**, *in silico* predictions of absorption, distribution, metabolism and elimination (ADME) parameters were made using the SwissADME web server.^51^ The SMILES of all compounds were used as input in the web server, and after the calculations, an output in .csv format was downloaded containing all of the data. Thus, gastrointestinal absorption (GI), log *P*_o/w_ (octanol/water), solubility using Ali class (soluble and moderately soluble), no brain–blood barrier (BBB) permeant, no P-glycoprotein (P-gp) substrate, and Drug-likeness with Lipinski, Ghose, and Veber rules were selected. The filtered compounds were drawn for molecular docking simulation using SMILES in Open Babel software.^32^ The Tanimoto coefficient was calculated using the best new benzothiazole compound and all 69 compounds from the series with Open Babel software.^32^

Molecular models of all compounds were created using MarvinSketch 19.21 (ChemAxon), considering a pH of 7.4, to estimate the best microspecies to simulate physiological conditions. All models were converted to 3D coordinates. Molecular geometries were optimized using the Steepest Descent method in Open Babel software.^32^

### Molecular Docking Studies Applied to a New 2-Aminobenzothiazole Core

The *Lm*PTR1 protein structure was subjected to a reprocessing in which solvents were removed, hydrogen and charges were added, and incomplete side chains were rebuilt using the Chimera UCSF program.^29^ The docking site was defined by a compound **1** binding cleft. A 25 Å^3^ grid box around the active site was used as the search space. Docking was performed through AutoDock Vina 1.1.2^52^ using the protein structure and the compounds obtained from the ZINC and PubChem databases. Every compound was evaluated by molecular docking studies using AutoDock Vina^43^ and by predicting the biological activity using the best QSAR model.

### Molecular Dynamic Simulations

All simulations were carried out using the GROMACS simulation package version 2021.2^30^ and Charmm36^53^ force field. The best compound and a chroman-4-One inhibitor (PDB ID 5L4N, ligand PDB ID 6QT(24)) had their topology built using CGenFF.^54^ All simulations were carried out with NADP.

The solvent properties were emulated using the TIP3P water model with a dodecahedron box large enough to allow for a minimum of 1.0 nm space from the protein to the box walls. The system charge was neutralized with the addition of ions at a physiological concentration of 0.15 mM. Geometry optimization of the solvated system was performed using the steepest descent algorithm (5000 steps), followed by equilibrating simulations with NVT (using 30 ns), simulated annealing (using 1 ns), and NPT (using 30 ns) ensembles. The temperature was kept at 300 K by coupling the system to a V-rescale thermostat (0.1 ps) in NVT and NPT, with the pressure kept constant at 1 bar using the Parinello–Rahman coupling algorithm. Furthermore, in simulated annealing, the system was gradually heated from 300 to 323.15 K in triplicate, using 5 points of annealing, over a period of 1 ns. A second equilibration using NPT was performed by restraining the protein backbone for 1 ns. Finally, unrestrained production simulations were conducted under the NPT ensemble at 300 K for 100 ns with a 2 fs time step.

To evaluate protein–ligand interactions, we analyzed the RMSD, RMSF (root mean square fluctuation), and number of hydrogen bonds (H-bond) between protein and ligands along the trajectory. The UCSF Chimera software^29^ was used to visualize the MD simulation, and plots were generated with xmgrace.^55^

### Clusterization

Applying a clustering method to the molecular trajectory was necessary after the molecular dynamics simulation. The aim of this analysis was to identify the most representative structure and determine if there were any noticeable variations in the system’s conformation. To achieve this aim, the hierarchical algorithm of agglomerative clustering was used, using an epsilon value of 2.0 Å, and performed in CPPTRAJ software.^56^ A cluster representing 99% of the trajectory for the PDB 6QT ligand was identified as well as a cluster representing 90% for the ZINC 72229720 ligand. The representative centroid of each cluster was then used to perform NCI calculations and semiempirical calculations of Δ*H*_binding_.

### Non-Covalent Interactions (NCI) Calculations

Using NCIPLOT^57^ software, non-covalent intermolecular interactions for ligands with PDB code 6QT and ZINC 72229720 were calculated between active site residues and the molecules of water within 3.0 Å around the ligand. For this purpose, the promolecular density was utilized.

### Quantum Chemical Calculation of Δ*H*_binding_

As the final step, we performed single-point quantum chemical calculations to determine the relative binding enthalpy (Δ*H*_binding_) for both simulated complexes, employing the semiempirical PM7 approach.^58^ This methodology included the application of the MOZYME^59^ linear scaling technique within the MOPAC package;^27^ default criteria for SCF convergence with a radius cutoff of 9 Å; utilization of a COSMO implicit solvent model with a dielectric constant of 78.4; and an effective radius of the solvent molecule set to 1.3 Å. The binding enthalpy was computed by using eq 2
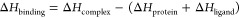
2

## Supporting Information Available

Alignment of *Lm*PTR1 and *Tb*PTR1 (Figure S1); QSAR model validation plots (Figure S2); 3D representation of docking results (Figures S3 and S4); ROC curve of molecular docking (Figure S5); 3D representation and plots of molecular dynamics simulation (Figure S6); 3D representation of overlaps of *Lm*PTR1 and *Tb*PTR1 (Figure S7); alignment of benzothiazole derivatives (Figure S8); representation of descriptors of the QSAR model (Table S1); docking results (score) (Table S2); Tanimoto similarity of ZINC 72229720 with benzothiazole compounds (Table S3); ADME properties of the best compounds (Table S4); and data set used in this study (Table S5) (PDF)

## Abbreviations

ADME: absorption–distribution–metabolism–excretion
AUC: area under the curve
BBB: brain–blood barrier
QQ: Coulomb descriptors
DHFR-TS: dihydrofolate reductase-thymidylate synthase
GI: gastrointestinal absorption
HB: hydrogen bonds
HF: hydrophobic
LNO: leave-N-out
LJ: Lennard-Jones equation
MLR: multiple linear regression
pIC_50_: negative log of inhibitory concentration
NADP: nicotinamide adenine dinucleotide phosphate
P-gp: glycoprotein
PDB: Protein Data Bank
PTR1: pteridine reductase 1
QSAR: quantitative structure–activity relationship
ROC: receiver operating characteristic
RMSD: root mean square deviation
SALI: structure landscape activity index
TS: Tanimoto similarities
