# Supplementary material for: In Silico Structural Insights and Potential Inhibitor Identification Based on the Benzothiazole Core for Targeting Leishmania major Pteridine Reductase 1
Source: ACS Omega. 2024 Dec 20;10(1):306–17. doi: 10.1021/acsomega.4c06146 (PMC11740253; doi:10.1021/acsomega.4c06146)
Supplement: Supplementary file 1 — ao4c06146_si_001.pdf [file ao4c06146_si_001.pdf]

In silico structural insights and potential inhibitor identification based on the benzothiazole core for targeting *Leishmania major* Pteridine Reductase 1

Jéssika de O. Viana[a], Karen C. Weber[a], Luiz E. G. da Cruz[a], Rhayane de O. Santos[a], Gerd B. Rocha[a], Alessandro K. Jordão[b], Euzébio G. Barbosa*[, b]

[a]Federal University of Paraíba, Department of Chemistry, João Pessoa, Brazil

[b]Federal University of Rio Grande do Norte, Department of Pharmacy, General Cordeiro de Farias Street, CEP: 59012-570 - Natal - RN, Brazil.

*Corresponding author, euzebio.guimaraes@ufrn.br

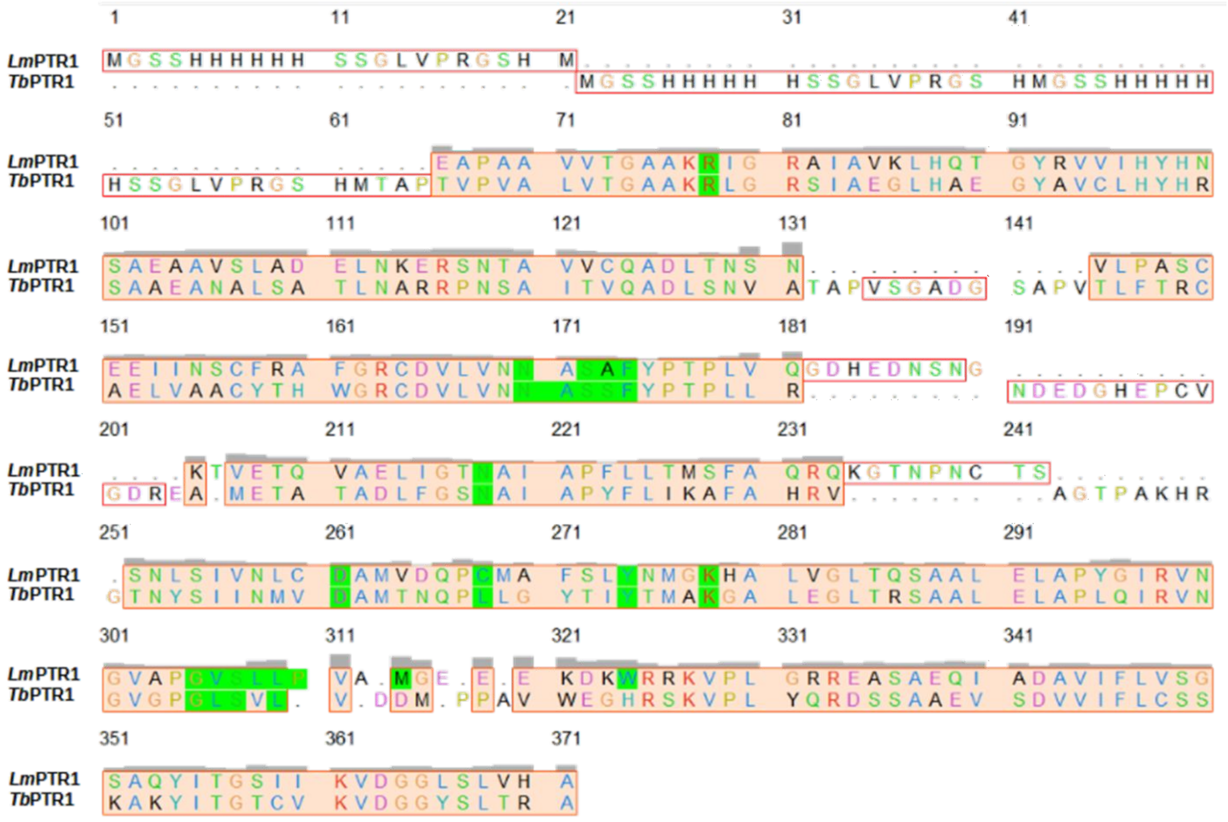

Figure S1. Alignment of the amino acid sequences of the enzymes *Lm*PTR1 and *Tb*PTR1, with the active site region being marked in green.

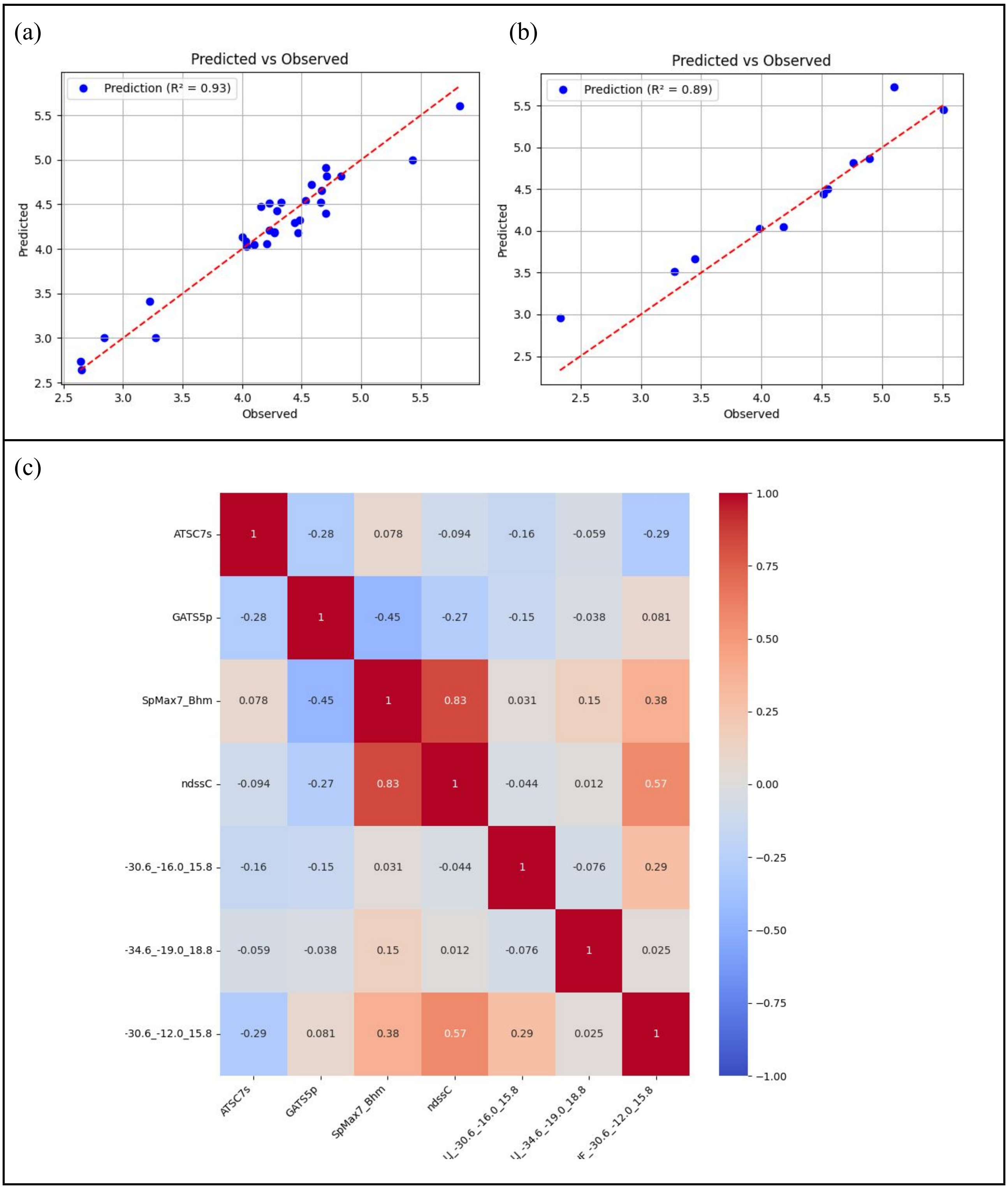

Figure S2. QSAR validation. (a) Observed *versus* predicted values of molecules in the training set. (b) Observed *versus* predicted values of molecules in the test set. (c) Heat map representation of the intercorrelation matrix of molecular descriptors.

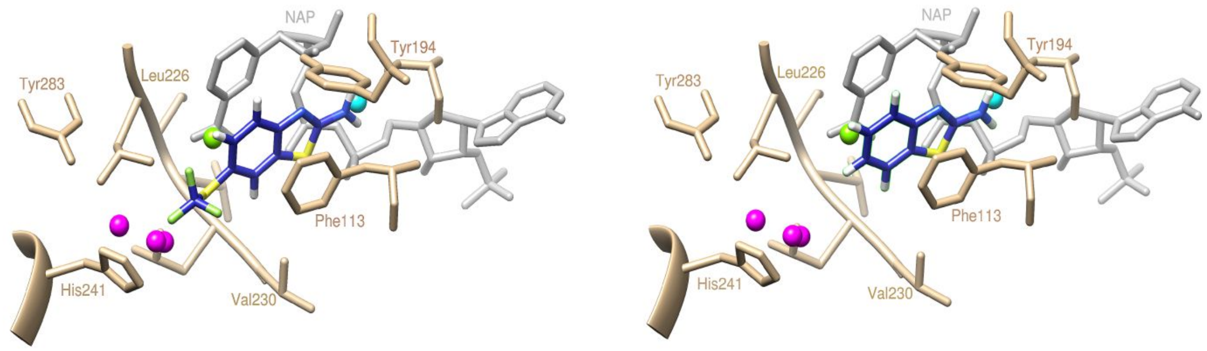

Compound 1
IC_{50} 1.9 μM

Compound 13
IC_{50} 1800 μM

Figure S3. Molecular representation of the 3D descriptors in the most potent (compound **1**) and the less potent (compound **12**) compounds of the benzothiazoles series. The tan color represents the protein, the gray color represents the NADP, pink sphere represents the LJ descriptors, and the green sphere represents the HF descriptor. For compounds, the dark blue color represents carbon, white represents hydrogen, yellow represents sulfur, and green represents fluorine atoms.

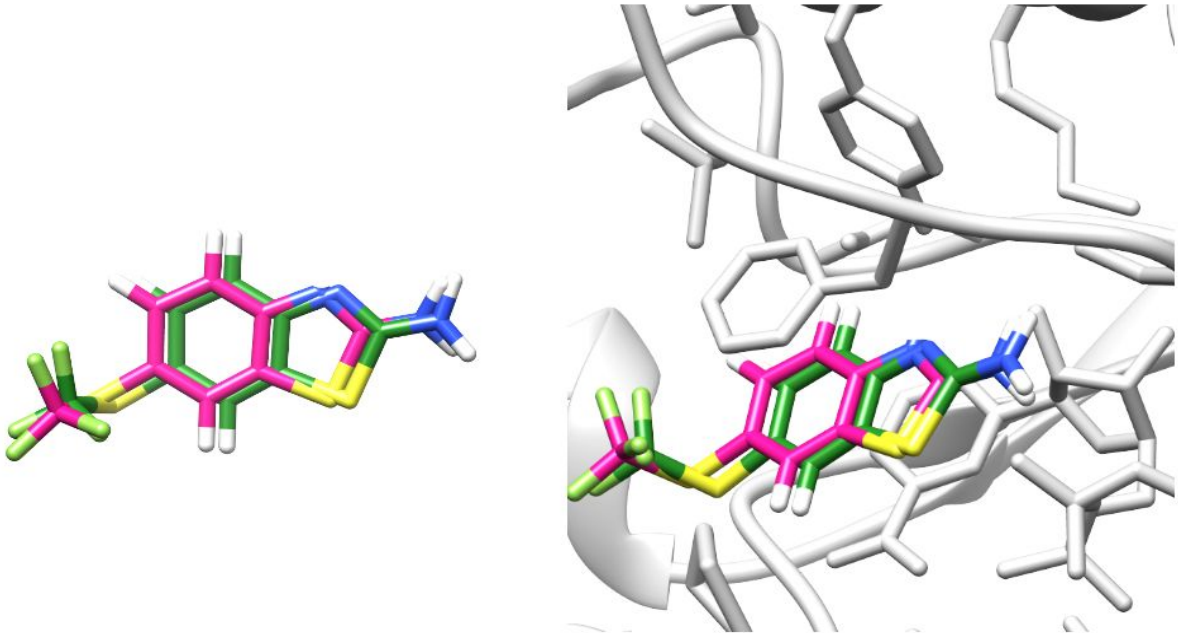

Figure S4. Overlay and binding mode of crystallized compound **1** (green) and simulated redocking compound **1** (pink) in the *Lm*PTR1 target.

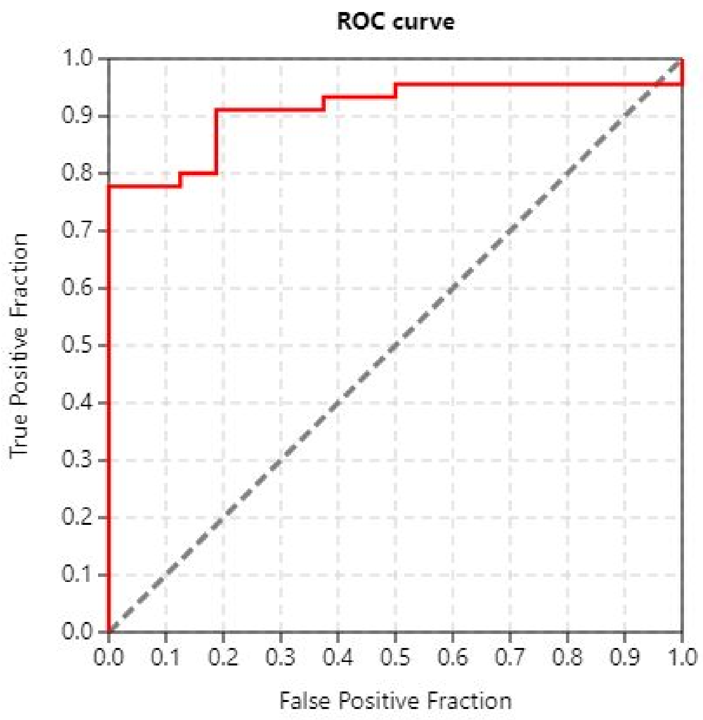

Figure S5. Representation of the validation of the molecular docking model using ROC.

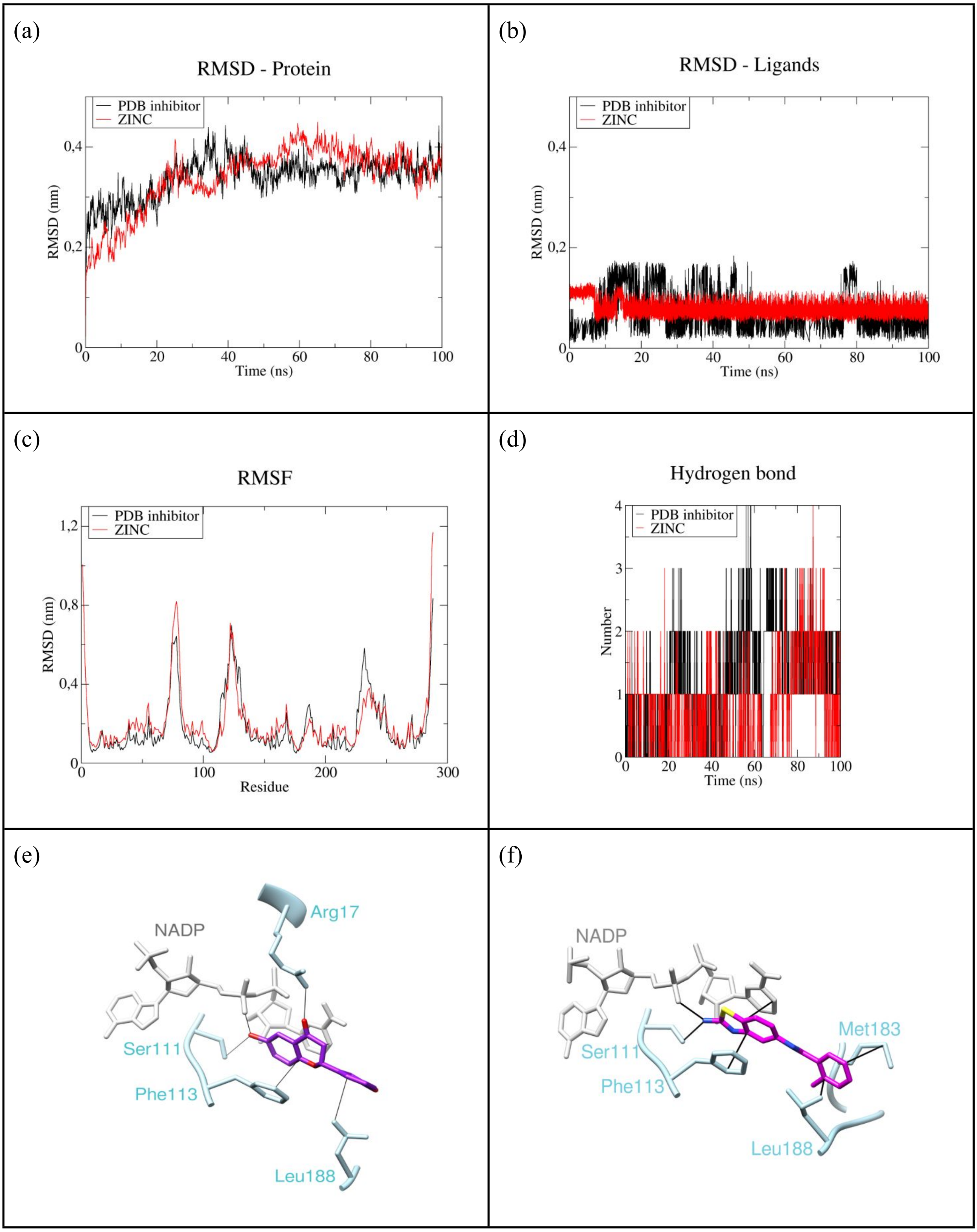

Figure S6. Result of molecular dynamics applied to complex systems: *Lm*PTR1-chroman-4-One (6QT) and *Lm*PTR1-ZINC 72229720. (a) RMSD of the protein throughout the simulation. (b) RMSD of the ligands throughout the simulation. (c) RMSF of the protein throughout the simulation. (d) Hydrogen bonds between *Lm*PTR1 and ligands. (e) Best cluster for 6QT ligand system. (f) Best cluster for ZINC 72229720 ligand system.

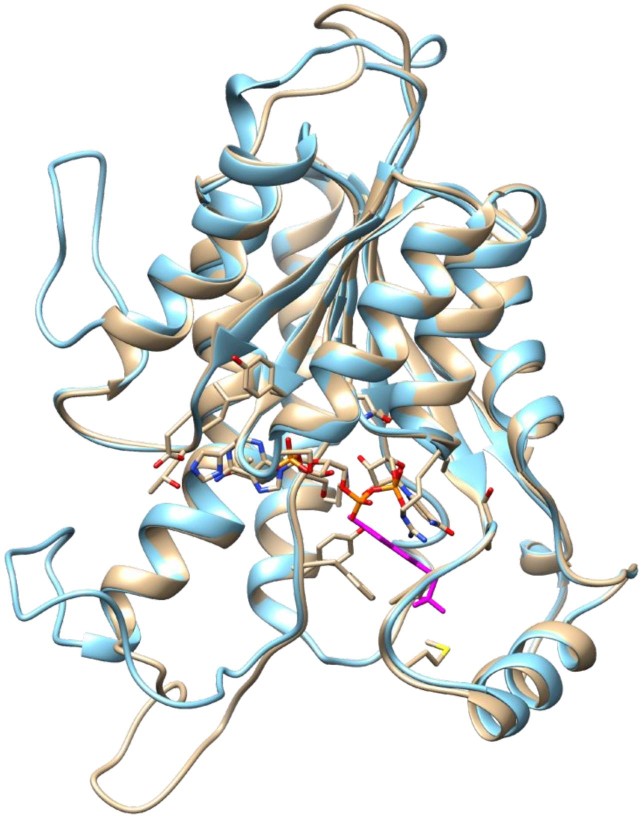

Figure S7. *Lm*PTR1 overlaps with *Tb*PTR1. The blue protein is reference to *L. major*, the gold protein is reference to *T. brucei* species. The red color is the NADP and the pink color is reference to compound **2b**.

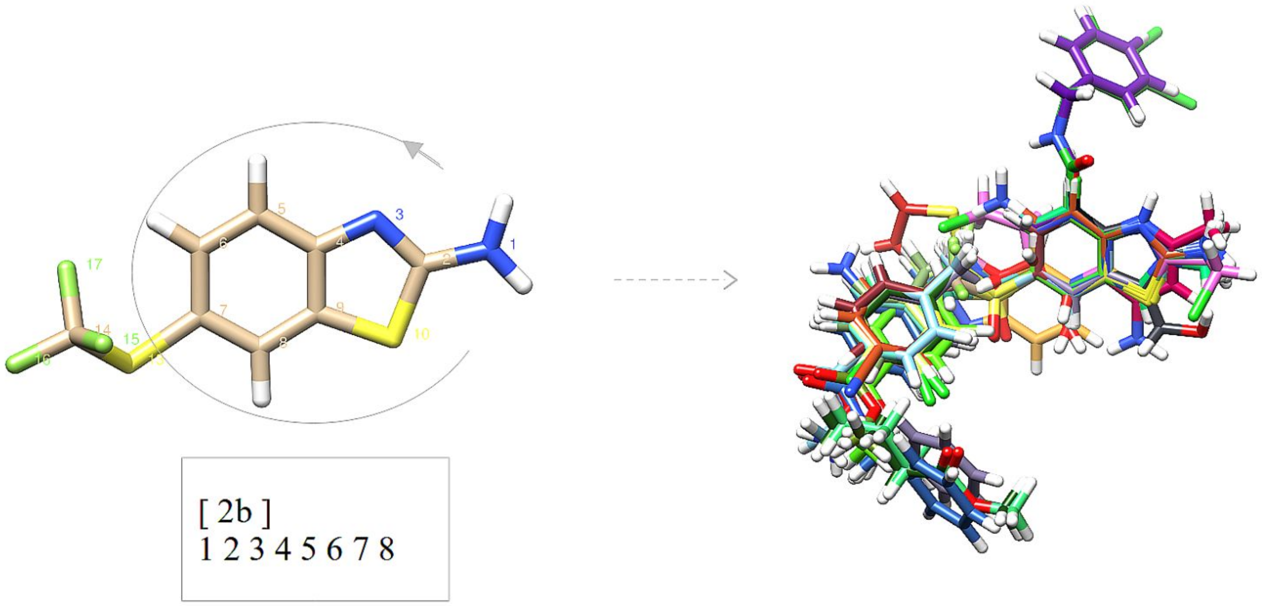

Figure S8. Automatic alignment scheme using *gmx confrms*, the module embedded in the GROMACS simulation package. Note the compound reference, the compound **1**, the serial number of the 2-aminobenzothiazole fragment and the study series compounds aligned.

Table S1. Matrix of representative descriptors of the hybrid QSAR model, showing a total of nine 2D descriptors (ndssC, SpMax7_Bhm, ATSC7s, GATS5p) and two 3D descriptors (LJ and HF), explained in 11 latent variables (LV).

LV	ndssC	SpMax7_Bhm	ATSC7s	GATS5p	LJ	HF
LV 1	6	2.65	9.67	1.05	0.65	0.47
LV 2	8	2.71	4.77	1.05	-0.03	0.47
LV 3	7	2.71	7.65	0.98	-0.14	0.47
LV 4	6	2.49	5.63	1.05	-0.01	0.73
LV 5	3	1.09	4.26	1.02	0	0.04
LV 6	5	2.96	6.99	1.12	0	0
LV 7	5	2.45	18.04	0.84	-0.07	0.46
LV 8	3	2.21	-8.22	1.06	-0.01	0
LV 9	4	2.16	23.29	1.07	-0.01	0.47
LV 10	8	2.87	47.54	0.81	0.21	1.01
LV 11	4	2.14	3.54	1.08	-0.01	0.81

Table S2. Molecular docking of the benzothiazoles series in *Lm*PTR1. The docking score is represented in kcal/mol.

Compound	SMILES	Score
**1**	Nc1nc2ccc(SC(F)(F)F)cc2s1	-9,7
**2**	Nc1nc2ccc(SCc3ccc(Cl)c(Cl)c3)cc2s1	-9
**3**	CS(=O)(=O)c1ccc2nc(N)sc2c1	-7,2
**4**	Nc1ccncc1	-4,8
**5**	Nc1nnc(s1)-c1cccc(c1)N(=O)=O	-5,3
**6**	Nc1nc2ccc(cc2s1)C(=O)NCc1ccccc1N	-9,4
**7**	Nc1nc2ccc(cc2s1)C(=O)NCc1ccccc1N(=O)=O	-9.0
**8**	NC1=NC2=CC=C(C=C2S1)C(=O)N(CC1=CC=CC=C1)CC1=CC=C(Cl)C(Cl)=C1	-6,3
**9**	Nc1nc2ccc(cc2s1)C(=O)NCc1ccc(Cl)c(Cl)c1	-9,3
**10**	Nc1nc2ccc(cc2s1)C(=O)NC(c1ccccc1)c1ccccc1	-9,4
**11**	Nc1nc2ccc(cc2s1)C(=O)NCc1ccc(cc1)C(=O)NCc1ccccc1	-9,3
**12**	Nc1nc2ccc(SCc3ccc(cc3)C#N)cc2s1	-8,7
**13**	COC(=O)c1ccc(CSc2ccc3nc(N)sc3c2)cc1	-8,8
**14**	COC(=O)C1CCN(CC1)C(=O)c1ccc(CSc2ccc3nc(N)sc3c2)cc1	-9,2
**15**	Nc1nc2ccc(SCc3ccc(cc3)C(=O)NCc3ccccc3)cc2s1	-9,1
**16**	CS(=O)(=O)c1ccc2nc(N)sc2c1	-8,3
**17**	COC(=O)c1ccc(CS(=O)(=O)c2ccc3nc(N)sc3c2)cc1	-8,4
**18**	COC(=O)C1CCN(CC1)C(=O)c1ccc(CS(=O)(=O)c2ccc3nc(N)sc3c2)cc1	-8,9
**19**	Nc1nc2ccc(cc2s1)C(=O)NCc1ccc(cc1)C#N	-9,4
**20**	Nc1nc2ccc(OC(F)(F)F)cc2s1	-7,4
**21**	COC(=O)c1ccc(CNC(=O)c2ccc3nc(N)sc3c2)cc1	-8,7

**22**	Nc1nc2c(O)cc(cc2s1)C(=O)NCc1ccccc1	-9,5
**23**	Nc1nc2c(cc(O)c(O)c2s1)C(=O)N(Cc1ccccc1)Cc1ccc(Cl)c(Cl)c1	-9,3
**24**	Nc1nc2c(cc(O)c(O)c2s1)C(=O)NCc1ccc(Cl)c(Cl)c1	-9,2
**25**	Nc1nc2c(cc(O)c(O)c2s1)C(=O)NCc1ccccc1	-9,2
**26**	CCC1=C(C(=NC(=N1)N)N)C2=CC=C(C=C2)Cl	-9,4
**27**	O=CC1=CNc2ccccc12	-7,1
**28**	C[N]1=Cc2ccc(N)cc2N1	-7
**29**	Cc1cc(N)c2ccccc2n1	-7,6
**30**	Oc1ccc(cc1)C(=O)c1ccc(F)cc1	-9,3
**31**	Nc1nnc(s1)-c1cnccc1N	-7
**32**	Nc1nnc(s1)-c1ccc2n[nH]nc2c1	-8,3
**33**	Nc1nnc(s1)C1=CC(=O)c2ccccc2O1	-8,8
**34**	Nc1nnc(CCC(=O)c2cccs2)s1	-7
**35**	Nc1nnc(CCC(=O)c2ccc(Cl)cc2)s1	-8,3
**36**	ClCCC(=O)Nc1nnc(CCCl)s1	-9,2
**37**	Nc1nc2ccccc2s1	-6,7
**38**	Nc1nc2c(Cl)cccc2s1	-6,6
**39**	Nc1nc2ccc(cc2s1)N(=O)=O	-7,1
**40**	Nc1nc2ccc(OCc3ccc(cc3)C#N)cc2s1	-9,1
**41**	COC(=O)c1ccc(COc2ccc3nc(N)sc3c2)cc1	-8,8
**42**	COC(=O)C1CCN(CC1)C(=O)c1ccc(COc2ccc3nc(N)sc3c2)cc1	-9,3
**43**	Nc1nc2ccc(SCc3ccccc3)cc2s1	-8,9
**44**	OC(=O)C1=CC=C2C=CNC2=C1	-8,9
**45**	CS(=O)(=O)c1ccc2nc(N)sc2c1	-7,4

**46**	O=C(CN1C(=O)c2ccccc2C1=O)Nc1nnc(CN2C(=O)c3ccccc3C2=O)s1	-9,3
**47**	Nc1nncs1	-4,3
**48**	Cc1nnc(N)s1	-6,7
**49**	Nc1nc2ccc(cc2s1)C(=O)NCCC1=CNC2=CC=CC=C12	-5,3
**50**	COc1ccc(CN(Cc2ccc(Cl)c(Cl)c2)C(=O)c2ccc3nc(N)sc3c2)cc1OC	-6,9
**51**	Nc1nc2ccc(OCc3ccc(Cl)c(Cl)c3)cc2s1	-6
**52**	Nc1nc2ccc(OCc3ccccc3)cc2s1	-6,7
**53**	CC[C@@H](C)c1nnc(N)s1	-5,9
**54**	Nc1nnc(s1)-c1ccccc1	-7,4
**55**	Nc1nnc(s1)-c1ccco1	-6,3
**56**	Nc1nnc(s1)-c1ccc(cc1)C#N	-6,2
**57**	CCOc1ccc(cc1)-c1nnc(N)s1	-7,4
**58**	NCCc1ccc(cc1)-c1nnc(N)s1	-6,8
**59**	Cc1cc(C)cc(c1)-c1nnc(N)s1	-7,2
**60**	Nc1nnc(C[C@@H]2NC(=O)NC2=O)s1	-6,8
**61**	Nc1nnc(CN2C(=O)c3ccccc3C2=O)s1	-5,9
**62**	Nc1nnc(s1)[C@H](O)c1ccccc1	-6,2
**63**	Nc1nnc(CCc2ccccc2)s1	-5,8
**64**	Nc1nnc(CCOc2ccc(Cl)cc2)s1	-5,5
**65**	Nc1nnc(CCCC(=O)c2ccccc2)s1	-6,4
**66**	Nc1nnc(s1)-c1cccc(NS(=O)(=O)c2cccc(c2)N(=O)=O)c1	-6,7
**67**	Nc1nnc(s1)-c1cccc(NS(=O)(=O)c2ccc(cc2)N(=O)=O)c1	-6,7
**68**	O=C(Nc1nnc(s1)-c1ccncc1)c1ccncc1	-7,3
**69**	Cc1ccc(cc1C)C(=O)Nc1nnc(s1)-c1ccc(C)c(C)c1	-7,5

Table S3. Tanimoto similarity calculated between the Zinc 72229720 and the benzothiazole compounds.

Compounds	Tanimoto similarity
**1**	0.663
**2**	0.836
**3**	0.157
**4**	0.160
**5**	0.496
**6**	0.463
**7**	0.463
**8**	0.463
**9**	0.484
**10**	0. 504
**11**	0.693
**12**	0.631
**13**	0.536
**14**	0.632
**15**	0.836
**16**	0.574
**17**	0.496

**18**	0.480
**19**	0.575
**20**	0.463
**21**	0.450
**22**	0.370
**23**	0.370
**24**	0.393
**25**	0.128
**26**	0.196
**27**	0.138
**28**	0.169
**29**	0.102
**30**	0.152
**31**	0.171
**32**	0.129
**33**	0.157
**34**	0.157
**35**	0.100
**36**	0.686

**37**	0.570
**38**	0.585
**39**	0.456
**40**	0.449
**41**	0.386
**42**	0.754
**43**	0.189
**44**	0.136
**45**	0.153
**46**	0.127
**47**	0.114
**48**	0.425
**49**	0.403
**50**	0.446
**51**	0.487
**52**	0.114
**53**	0.141
**54**	0.114
**55**	0.132

**56**	0.152
**57**	0.136
**58**	0.136
**59**	0.123
**60**	0.176
**61**	0.133
**62**	0.151
**63**	0.140
**64**	0.150
**65**	0.170
**66**	0.167
**67**	0.163
**68**	0.124
**69**	0.067

Table S4. Comparison of IC_{50} values and ADME profiles of inhibitor 6QT, ZINC 72229720, and compound **1**. Lipophilicity was determined through 1-octanol/water solvent partitioning coefficients ($LogP_{o/w}$), gastrointestinal absorption (GI), water solubility, blood-brain barrier penetration, P-glycoprotein (P-gp) interaction, Lipinski's rules, and lead-likeness profile.

Compound	IC_{50} µM	$LogP_{o/w}$	GI	Water solubility	BBB	P-gp substrate	Lipinski	drug-likeness
6QT inhibitor	57	2.05	high	soluble	Yes	Yes	0	0
compound **1**	1.9	3.26	high	moderately soluble	No	No	0	1
Zinc 72229720	0.095*	2.91	high	soluble	No	No	0	0

* predicted (pIC_{50}) using the QSAR model.

Table S5. Dataset used in the work, using a total of 69 benzothiazole compounds tested for the *Lm*PTR1 in µM.

Name	Compound	Smiles	Activity µM	pIC_{50}	Reference	Qsar model
**1**	2b	Nc1nc2ccc(SC(F)(F)F)cc2s1	1.9	5.72	[13]	Test

**2**	2d	Nc1nc2ccc(SCc3ccc(Cl)c(Cl)c3)cc2s1	10	5.00	[13]	Training
**3**	4c	CS(=O)(=O)c1ccc2nc(N)sc2c1	212	3.67	[12]	Test
**4**	6a	Nc1ccncc1	1900	2.72	[12]	Training
**5**	8b	Nc1nnc(s1)-c1cccc(c1)N(=O)=O	Inactive	-	[12]	Not included
**6**	4k	Nc1nc2ccc(cc2s1)C(=O)NCc1ccccc1N	64.7	4.19	[13]	Training
**7**	4j	Nc1nc2ccc(cc2s1)C(=O)NCc1ccccc1N(=O)=O	3.51	5.45	[13]	Test
**8**	4p	NC1=NC2=CC=C(C=C2S1)C(=O)N(CC1=CC=CC=C1)CC1=CC=C(Cl)C(Cl)=C1	Inactive	-	[13]	Not included
**9**	4d	Nc1nc2ccc(cc2s1)C(=O)NCc1ccc(Cl)c(Cl)c1	15.3	4.82	[13]	Test
**10**	4l	Nc1nc2ccc(cc2s1)C(=O)NC(c1ccccc1)c1ccccc1	88.5	4.05	[13]	Training
**11**	4h	Nc1nc2ccc(cc2s1)C(=O)NCc1ccc(cc1)C(=O)NCc1ccccc1	18.9	4.72	[13]	Training
**12**	2e	Nc1nc2ccc(SCc3ccc(cc3)C#N)cc2s1	12.4	4.91	[13]	Training
**13**	2f	COC(=O)c1ccc(CSc2ccc3nc(N)sc3c2)cc1	47.6	4.32	[13]	Training
**14**	2g	COC(=O)C1CCN(CC1)C(=O)c1ccc(CSc2ccc3nc(N)sc3c2)cc1	15.1	4.82	[13]	Training
**15**	2h	Nc1nc2ccc(SCc3ccc(cc3)C(=O)NCc3ccccc3)cc2s1	30	4.52	[13]	Training
**16**	3a	CS(=O)(=O)c1ccc2nc(N)sc2c1	32.9	4.48	[13]	Training
**17**	3f	COC(=O)c1ccc(CS(=O)(=O)c2ccc3nc(N)sc3c2)cc1	65.9	4.18	[13]	Training
**18**	3g	COC(=O)C1CCN(CC1)C(=O)c1ccc(CS(=O)(=O)c2ccc3nc(N)sc3c2)cc1	50	4.30	[13]	Training
**19**	4e	Nc1nc2ccc(cc2s1)C(=O)NCc1ccc(cc1)C#N	73.3	4.13	[13]	Training
**20**	1b	Nc1nc2ccc(OC(F)(F)F)cc2s1	94.1	4.03	[13]	Training
**21**	4f	COC(=O)c1ccc(CNC(=O)c2ccc3nc(N)sc3c2)cc1	36	4.44	[13]	Test
**22**	4s	Nc1nc2c(O)cc(cc2s1)C(=O)NCc1ccccc1	2.48	5.61	[13]	Training
**23**	5a	Nc1nc2c(cc(O)c(O)c2s1)C(=O)N(Cc1ccccc1)Cc1ccc(Cl)c(Cl)c1	15.2	4.82	[13]	Training
**24**	5b	Nc1nc2c(cc(O)c(O)c2s1)C(=O)NCc1ccc(Cl)c(Cl)c1	36.8	4.43	[13]	Training
**25**	5c	Nc1nc2c(cc(O)c(O)c2s1)C(=O)NCc1ccccc1	30.1	4.52	[13]	Training

**26**	Pyrimethamine	CCC1=C(C(=NC(=N1)N)N)C2=CC=C(C=C2)Cl	13.6	4.87	[13]	Test
**27**	28a	O=CC1=CNc2ccccc12	1100	2.96	[12]	Test
**28**	35a	C[N]1=Cc2ccc(N)cc2N1	1000	3.00	[12]	Training
**29**	38a	Cc1cc(N)c2ccccc2n1	390	3.41	[12]	Training
**30**	53a	Oc1ccc(cc1)C(=O)c1ccc(F)cc1	2300	2.64	[12]	Training
**31**	7b	Nc1nnc(s1)-c1cnccc1N	31	4.51	[12]	Training
**32**	14b	Nc1nnc(s1)-c1ccc2n[nH]nc2c1	309	3.51	[12]	Test
**33**	15b	Nc1nnc(s1)C1=CC(=O)c2ccccc2O1	22	4.66	[12]	Training
**34**	21b	Nc1nnc(CCC(=O)c2cccs2)s1	29	4.54	[12]	Training
**35**	22b	Nc1nnc(CCC(=O)c2ccc(Cl)cc2)s1	89	4.05	[12]	Training
**36**	28b	ClCCC(=O)Nc1nnc(CCCl)s1	93	4.03	[12]	Test
**37**	1c	Nc1nc2ccccc2s1	1800	2.74	[12]	Test
**38**	2c	Nc1nc2c(Cl)cccc2s1	1000	3.00	[12]	Training
**39**	3c	Nc1nc2ccc(cc2s1)N(=O)=O	40	4.40	[12]	Training
**40**	1e	Nc1nc2ccc(OCc3ccc(cc3)C#N)cc2s1	86.5	4.06	[13]	Training
**41**	1f	COC(=O)c1ccc(COc2ccc3nc(N)sc3c2)cc1	66.6	4.18	[13]	Training
**42**	1g	COC(=O)C1CCN(CC1)C(=O)c1ccc(COc2ccc3nc(N)sc3c2)cc1	31.9	4.50	[13]	Test
**43**	2c	Nc1nc2ccc(SCc3ccccc3)cc2s1	81	4,09	[13]	Training
**44**	29a	OC(=O)C1=CC=C2C=CNC2=C1	Inactive	-	[12]	Not included
**45**	5c	CS(=O)(=O)c1ccc2nc(N)sc2c1	50	4.30	[12]	Training
**46**	29b	O=C(CN1C(=O)c2ccccc2C1=O)Nc1nnc(CN2C(=O)c3ccccc3C2=O)s1	116	3.94	[12]	Training
**47**	4a	Nc1nncs1	5600	2.25	[12]	Training
**48**	1b	Cc1nnc(N)s1	Inactive	-	[12]	Not included
**49**	4o	Nc1nc2ccc(cc2s1)C(=O)NCCC1=CNC2=CC=CC=C12	Inactive	-	[13]	Not

						included
**50**	4r	COc1ccc(CN(Cc2ccc(Cl)c(Cl)c2)C(=O)c2ccc3nc(N)sc3c2)cc1OC	Inactive	-	[13]	Not included
**51**	1d	Nc1nc2ccc(OCc3ccc(Cl)c(Cl)c3)cc2s1	Inactive	-	[13]	Not included
**52**	1c	Nc1nc2ccc(OCc3ccccc3)cc2s1	Inactive	-	[13]	Not included
**53**	3b	CC[C@@H](C)c1nnc(N)s1	Inactive	-	[12]	Not included
**54**	4b	Nc1nnc(s1)-c1ccccc1	Inactive	-	[12]	Not included
**55**	5b	Nc1nnc(s1)-c1ccco1	Inactive	-	[12]	Not included
**56**	9b	Nc1nnc(s1)-c1ccc(cc1)C#N	Inactive	-	[12]	Not included
**57**	10b	CCOc1ccc(cc1)-c1nnc(N)s1	Inactive	-	[12]	Not included
**58**	11b	NCCc1ccc(cc1)-c1nnc(N)s1	Inactive	-	[12]	Not included
**59**	13b	Cc1cc(C)cc(c1)-c1nnc(N)s1	Inactive	-	[12]	Not included
**60**	16b	Nc1nnc(C[C@@H]2NC(=O)NC2=O)s1	Inactive	-	[12]	Not included
**61**	17b	Nc1nnc(CN2C(=O)c3ccccc3C2=O)s1	Inactive	-	[12]	Not included
**62**	18b	Nc1nnc(s1)[C@H](O)c1ccccc1	Inactive	-	[12]	Not included
**63**	19b	Nc1nnc(CCc2ccccc2)s1	Inactive	-	[12]	Not included

**64**	20b	Nc1nnc(CCOc2ccc(Cl)cc2)s1	Inactive	-	[12]	Not included
**65**	23b	Nc1nnc(CCCC(=O)c2ccccc2)s1	Inactive	-	[12]	Not included
**66**	24b	Nc1nnc(s1)-c1cccc(NS(=O)(=O)c2cccc(c2)N(=O)=O)c1	Inactive	-	[12]	Not included
**67**	25b	Nc1nnc(s1)-c1cccc(NS(=O)(=O)c2ccc(cc2)N(=O)=O)c1	Inactive	-	[12]	Not included
**68**	26b	O=C(Nc1nnc(s1)-c1ccncc1)c1ccncc1	Inactive	-	[12]	Not included
**69**	27b	Cc1ccc(cc1C)C(=O)Nc1nnc(s1)-c1ccc(C)c(C)c1	Inactive	-	[12]	Not included
